# DNA delivered by lipid nanoparticles induces CD8^+^ T cell–dependent antitumor responses and enhances anti–PD-L1 therapy

**DOI:** 10.1172/jci.insight.197404

**Published:** 2026-03-23

**Authors:** Seoyun Yum, Alba Rodríguez-Garcia, Joan Castellsagué, Marta Giménez-Alejandre, Guillem Colell, Salut Colell, Teresa Lobo-Jarne, Mark A. LaRue, Michael A. Minnier, Mustafa N. Yazicioglu, Rui Zhang, Xavier M. Anguela, Ali Nahvi, Matthew C. Walsh, Sean M. Armour, Sonia Guedan, Pedro J. Cejas

**Affiliations:** 1 Discovery Group, Spark Therapeutics Inc., Philadelphia, Pennsylvania, USA.; 2 Cellular Immunotherapies for Cancer Group, Institut d’Investigacions Biomèdiques August Pi i Sunyer (IDIBAPS), Barcelona, Spain.

**Keywords:** Immunology, Oncology, Adaptive immunity, Cancer immunotherapy, T cells

## Abstract

Immune checkpoint inhibitors (ICIs) have reshaped the treatment landscape of several cancer types. However, their effectiveness remains limited to a subset of patients, in part due to insufficient preexisting antitumor immunity. In this study, we hypothesized that intracellular delivery of noncoding dsDNA encapsulated in lipid nanoparticles (DNA-LNPs), which have recently been demonstrated to activate both STING and absent in melanoma 2 (AIM2) pathways, could enhance antitumor immune responses and potentiate ICI therapy. Using multiple animal models of cancer, including hepatocellular carcinoma, acute myeloid leukemia, melanoma, and melanoma lung metastasis, we show that DNA-LNP treatment triggered strong cytokine induction and robust CD8^+^ T cell recruitment to the tumor microenvironment. This immune activation mediated potent CD8^+^ T cell–dependent antitumor effects and prolonged animal survival across multiple models. Notably, empty LNPs did not elicit potent cytokine elevation or antitumor effects, suggesting that these responses are triggered by the activation of cytosolic DNA-sensing pathways. Moreover, DNA-LNPs synergized with anti–PD-L1, substantially extending animal survival in both ICI-responsive and ICI-resistant tumor models. These findings position DNA-LNPs as a promising immunotherapy strategy, either alone or in combination with ICI therapies, to enhance antitumor immunity across diverse cancer types.

## Abstract 2

We used various animal tumor models to demonstrate that delivery of DNA in nanoparticles boost the immune system’s attack on tumors and improves cancer immunotherapy effectiveness.

## Graphical abstract

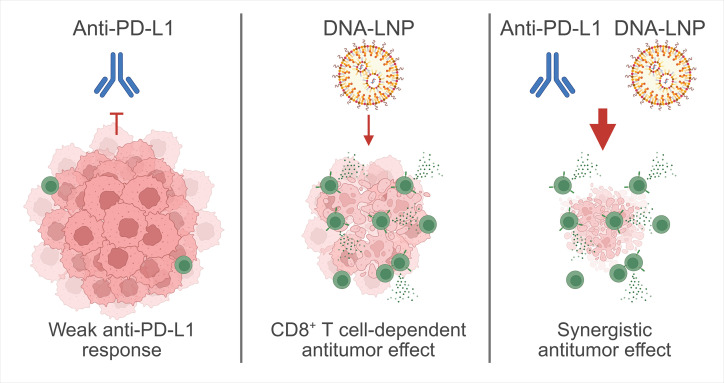

## Introduction

The immune system can identify and destroy cancer cells, but the tumor’s immunosuppressive mechanisms often inhibit these protective immune responses. Therapy with immune checkpoint inhibitors (ICIs), which aim at reverting the immune suppression, can mediate long-term responses, especially in a subset of tumors with numerous genetic mutations that are infiltrated with neoantigen-specific T cells (i.e., hot tumors). ICI therapy has revolutionized cancer treatment, highlighting the power of T cells in controlling solid tumors. However, a majority of patients fail to respond to ICI therapy (i.e., cold tumors) or relapse after a certain period of time (1). To unleash the antitumor effect of ICIs in cold tumors, multiple clinical trials are evaluating combinations of ICIs with immunostimulatory drugs to increase the number and activity of immune cells within the tumor.

Activating the STING pathway is one of the strategies explored to make tumors more responsive to immunotherapies. STING induces type I IFNs and cytokines upon binding to cyclic GMP-AMP, which is produced when cytosolic DNA is detected by cGAS (2, 3). In addition to detecting pathogen-derived cytosolic DNA, cGAS has been shown to spontaneously induce cancer immune surveillance by detecting increased cancer-derived cytosolic DNA (4). STING agonists, which further boost pathway activity, have shown antitumor effects in preclinical models and are being evaluated in clinical trials, alone or in combination with immune checkpoint blockade (5). However, the first STING agonist tested in a clinical trial, ADU-S100, showed poor pharmacokinetics and limited therapeutic effects, suggesting a need for more effective immune activation within tumors (6).

Simultaneously activating multiple innate immune signaling pathways is another strategy to potentiate immune stimulation (7). The pathways include multiple sensors that respond to diverse pathogen- or damage-associated molecular patterns and elicit distinct signaling cascades to induce immune responses. Drugs activating pathways such as TLRs, retinoic acid–inducible gene I (RIG-I), NLR family pyrin domain containing 3 (NLRP3), or STING are being evaluated in clinical trials, and the TLR7 agonist imiquimod is approved for treating basal cell carcinoma (8, 9). Combining these pathway activators enhanced immune responses and induced synergistic antitumor effects in multiple tumor models (10–16). Of note, tumor-treating fields, an approved alternating electric field therapy for glioblastoma, induce antitumor effects by activating both STING and AIM2 pathways (17).

Recent publications have reported the immunostimulatory properties of DNA encapsulated in lipid nanoparticles (DNA-LNPs) in the context of gene therapy, vaccine development, and cancer treatment, including their role as potent immune activators in tumor models (18–20). We recently reported that LNPs encapsulating DNA plasmids induce cytokines including type I IFNs, IL-18, and IFN-γ in mice by activating both STING and AIM2 pathways (21). Since these cytokines are critical for inducing antitumor immune responses (22, 23), we evaluated the antitumor effects of noncoding DNA-LNPs in mouse cancer models including hepatocellular carcinoma (HCC), acute myeloid leukemia, melanoma, and melanoma lung metastasis. Here, we demonstrate that DNA-LNPs induce protective CD8^+^ T cell–dependent antitumor responses across multiple mouse cancer models and act synergistically to boost effectiveness of anti–PD-L1 treatment in both ICI-responsive and ICI-resistant tumor models. These findings highlight DNA-LNP as a cancer immunotherapy with potential to broaden the population of patients who respond to ICIs.

## Results

### DNA delivered by LNPs induces an antitumor effect in mouse models of HCC.

HCC is an optimal target for DNA-LNP therapy given that standard LNP formulations mainly accumulate in the liver after i.v. administration in both mice and humans (24, 25). We hypothesized that DNA delivery by LNPs to the liver could trigger an innate immune response leading to tumor control.

**Figure 1:**
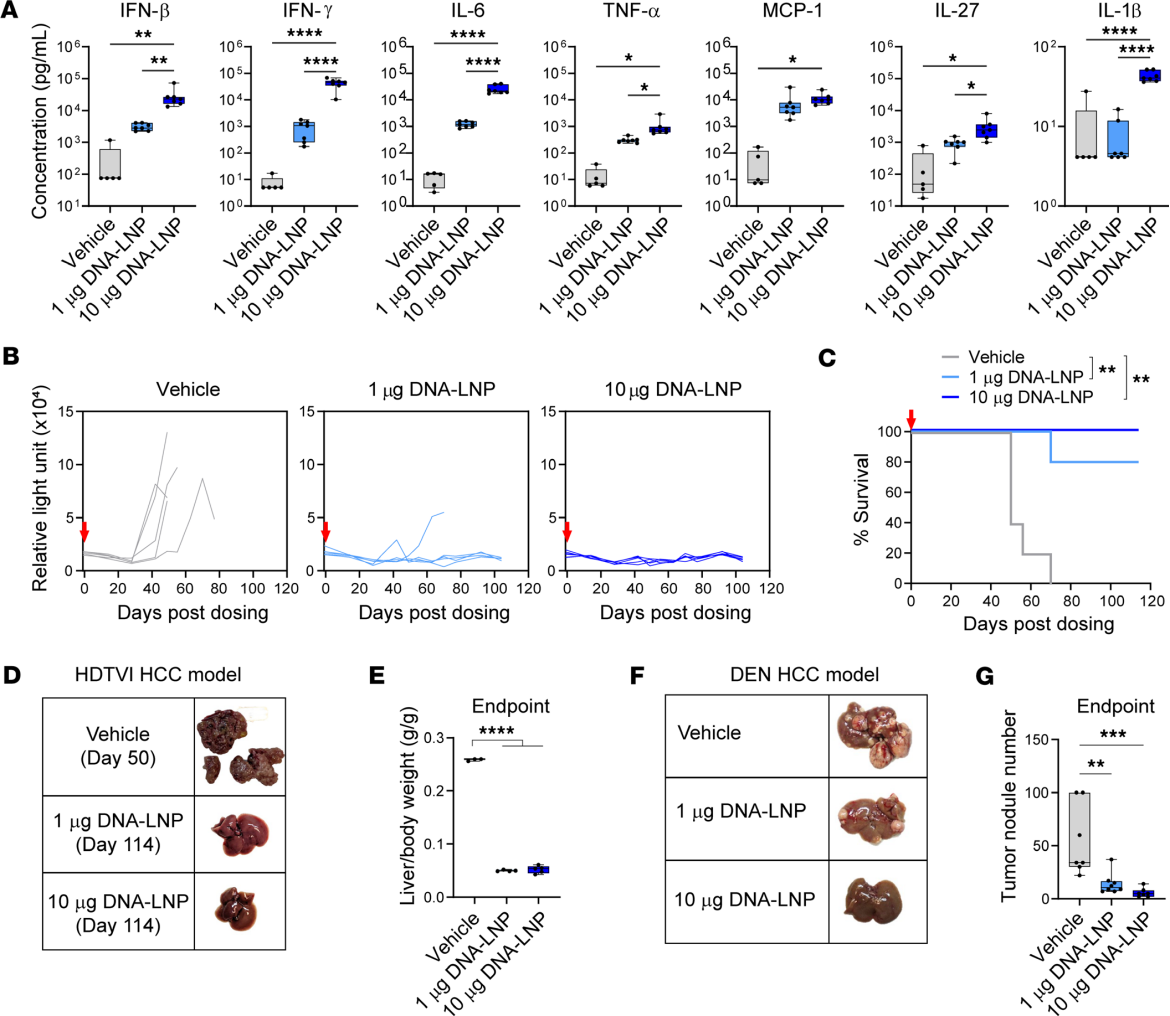
DNA-LNP induces a robust antitumor effect in HCC mouse models. (**A**–**E**) HCC was induced by HDTVI of plasmids encoding GLuc, c-MET, β-catenin (ΔN90), and HSB2. Mice received a single dose of DNA-LNP (i.v.) 3 weeks after HCC induction. (**A**) Serum cytokine levels 4 hours after dosing. (**B**) Tumor growth indicated by serum GLuc activity. (**C**) Survival of HCC-bearing mice. (**D**) Representative images of liver and (**E**) liver/body weight ratio at endpoints: day 50 for the vehicle and day 114 for the DNA-LNP groups. (**F** and **G**) HCC was induced by DEN administration; 36 weeks later, mice received a single dose of DNA-LNP (i.v.). (**F**) Representative images of the liver and (**G**) tumor nodule count at 6 weeks after treatment. Min-to-max whiskers are shown in box and whiskers plot. Survival data were analyzed by log-rank (Mantel-Cox) tests, and other data were analyzed by 1-way ANOVA with Tukey’s multiple-comparison test. *P < 0.05, **P < 0.01, ***P < 0.001, and ****P < 0.0001.

To test this hypothesis, we first employed a genetically induced mouse model of HCC, driven by the coexpression of c-MET and a constitutively active form of β-catenin (ΔN90). *Gaussia* luciferase (GLuc) was also expressed for serum-based tumor tracking (26). To express these transgenes in the liver, plasmids encoding oncogenes, GLuc, and the Sleeping Beauty transposase (HSB2) were administered by hydrodynamic tail vein injection (HDTVI). DNA-LNPs were i.v. administered to these mice 3 weeks after tumor induction. Supporting our hypothesis, cytokine analysis in serum 4 hours after treatment revealed a dose-dependent elevation in serum levels of type I IFN (IFN-β); proinflammatory cytokines such as TNF-α, IL-6, and IL-27; and the chemokine CCL2 (MCP-1), all of them indicative of cGAS-STING activation. IFN-γ, although not a direct product of cGAS-STING activation, was also prominently induced upon DNA-LNP treatment. IL-1β, indicative of inflammasome pathway activation, was detectably elevated in the higher-dose treatment group (10 μg) (Figure 1A). This cytokine elevation occurred regardless of tumor presence, as shown in mice that did not receive the plasmids containing the oncogenes (Supplemental Figure 1A; supplemental material available online with this article; https://doi.org/10.1172/jci.insight.197404DS1).

Importantly, a single dose of DNA-LNP resulted in potent tumor control, as reflected by reduced GLuc serum levels in treated mice (Figure 1B). No GLuc elevation in serum was observed in mice that did not receive the plasmids containing the oncogenes, confirming that GLuc levels correlated with increasing liver tumor burden (Supplemental Figure 1B). This control in tumor progression by DNA-LNP administration resulted in a marked extension in the survival of treated mice (Figure 1C). Although mice treated with vehicle (PBS) had a mean survival of 50 days after dosing, DNA-LNP–treated mice survived beyond 100 days. At their respective study endpoints, vehicle-treated mice (day 50) exhibited clear signs of liver tumor burden at the macroscopic level, whereas livers from DNA-LNP–treated mice (day 114) appeared healthy (Figure 1D). This was consistent with reduced liver/body weight ratios in the DNA-LNP groups (Figure 1E).

To validate our findings in an alternative model, we utilized a chemically induced HCC mouse model based on the genotoxic carcinogen diethylnitrosamine (DEN), which drives hepatocarcinogenesis through direct DNA damage, more closely resembling the mutational processes observed in patients (27). Specifically, HCC was induced by administering DEN to 14-day-old mice, and a single dose of DNA-LNP was administered 9 months after induction. DNA-LNP administration led to a marked decrease in the number of tumor nodules as assessed 6 weeks after treatment (Figure 1, F and G), further supporting our findings in the genetically induced model. In both HCC models, transient, dose-dependent body weight loss was observed after DNA-LNP treatment (Supplemental Figure 1, C and D). This body weight loss occurred regardless of tumor presence, as shown in mice that did not receive the plasmids containing the oncogenes (Supplemental Figure 1C).

**Figure 2:**
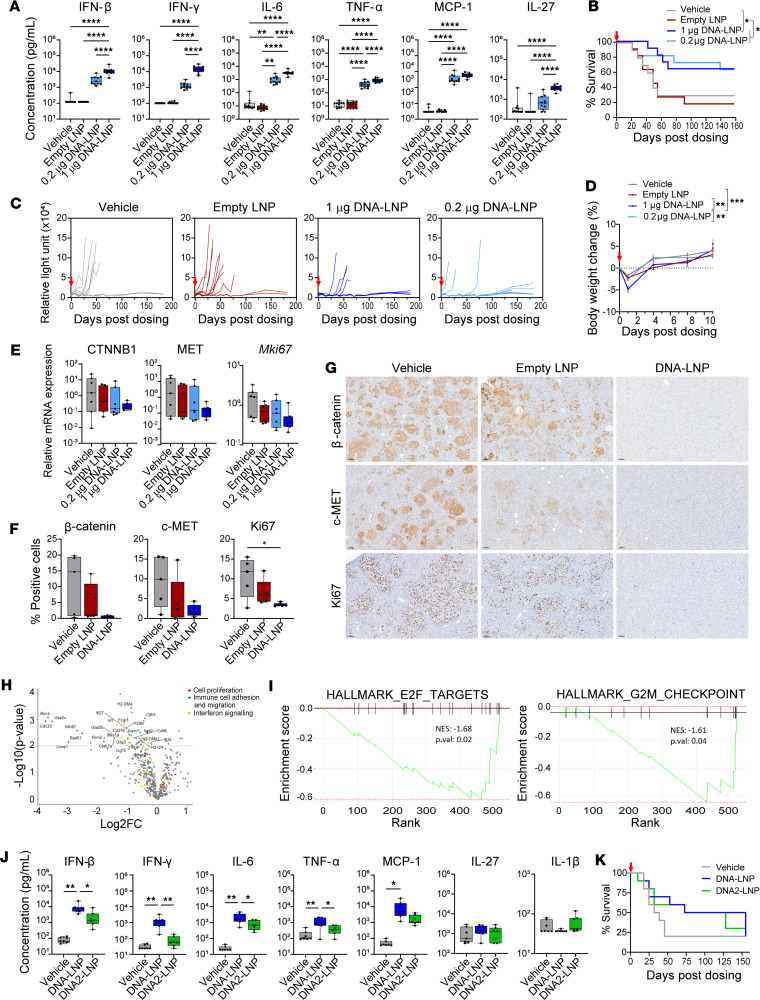
DNA delivered by LNP mediates the antitumor effect in HCC mouse model. HCC was induced by HDTVI of plasmids encoding GLuc, c-MET, β-catenin, and HSB2. (**A**–**I**) Mice received a single i.v. dose of DNA-LNP or empty LNP 3 weeks after HCC induction. Empty LNP contains an equivalent amount of empty LNP as 1 μg of DNA-LNP. (**A**) Serum cytokine levels 4 hours after dosing. (**B**) Survival of HCC-bearing mice. (**C**) Tumor growth indicated by serum GLuc activity. (**D**) Body weight loss measured over time. *P* value is compared at 1 day after LNP administration. (**E**) qRT-PCR analysis of oncogenes and ki67 at day 14 after treatment. (**F**) Quantification and (**G**) representative images (10×) of IHC analysis of oncogenes and Ki67 in liver sections at day 14 after treatment with 1 μg of DNA-LNP. (**H**) Volcano plot of differentially expressed genes and (**I**) GSEA in livers from mice treated with 1 μg of DNA-LNP as compared with vehicle at day 14 after treatment and measured by NanoString. (**J** and **K**) Mice received a single i.v. dose of 1 μg of DNA-LNP or DNA2-LNP 4 weeks after HCC induction. (**J**) Serum cytokine levels 4 hours after dosing. (**K**) Survival of HCC-bearing mice. Min-to-max whiskers are shown in the box and whiskers plot. Survival data were analyzed by log-rank (Mantel-Cox) tests, and other data were analyzed by 1-way ANOVA with Tukey’s multiple-comparison test. *P < 0.05, **P < 0.01, and ****P < 0.0001.

To investigate the key determinants of DNA-LNP–induced antitumor activity, we compared the in vivo activity of noncoding DNA-LNPs with LNPs lacking the DNA payload (empty LNPs) in the genetically induced HCC mouse model. No cytokine response (Figure 2A), antitumor efficacy (Figure 2, B and C), or body weight loss (Figure 2D) were triggered by empty LNPs, confirming that these effects were specifically mediated by cytosolic DNA delivered by LNPs. To further refine the therapeutic window, we tested a lower dose of 0.2 μg DNA-LNP and observed a milder elevation in serum cytokines, following a dose-dependent trend (Figure 2A), but conferred a survival benefit comparable to the 1 μg dose (Figure 2B) without the associated body weight loss (Figure 2D). Systemic cytokine levels at this dose were transiently elevated but returned to baseline within 24 hours (Supplemental Figure 2A). Additionally, 3 weekly doses of 0.2 μg DNA-LNP did not enhance the antitumor effect compared with a single dose (Supplemental Figure 2, B and C). These results suggest that even a low, single dose of DNA-LNP is sufficient to elicit a robust and durable antitumor effect.

In line with the observed antitumor activity, analysis of liver tissue 14 days after dosing revealed a reduction in the expression of the oncogenes cMET and β-catenin, as well as the proliferation marker Ki67 in DNA-LNP–treated mice as compared with the groups treated with vehicle or empty LNPs (Figure 2, E–G). These findings were confirmed at both the transcriptional level by RT-PCR (Figure 2E) and at the protein level by IHC (Figure 2, F and G). NanoString gene expression analysis performed at the same time point revealed downregulation of genes involved in pathways related to cell proliferation, immune cell adhesion and migration, and IFN signaling in DNA-LNP–treated mice (Figure 2H). We also performed gene set enrichment analysis (GSEA) of genes differentially expressed in DNA-LNP–treated mice using the Hallmark gene sets. This analysis revealed downregulation in genes associated with pathways linked to HCC tumor progression such as G2M checkpoints or E2F targets (Figure 2I) (28–30). Since our transcriptomic analysis used bulk liver homogenates, the NanoString data may reflect mixed cellular contributions. Nevertheless, taken together, these data suggest a reduction of tumor cells in the liver.

To assess whether DNA payload size and sequence specificity influence antitumor responses, we substituted the 5.1 kb noncoding DNA plasmid used to make DNA-LNPs with a smaller (1.3 kb) noncoding plasmid with a distinct sequence (DNA2). DNA2-LNP elicited a milder cytokine response profile compared with DNA-LNP (Figure 2J), but both DNA-LNPs demonstrated a similar survival benefit (Figure 2K). Together, these results demonstrate that LNP-mediated DNA delivery induces a robust innate immune response, antitumor effect, and extended survival in HCC mouse models.

### DNA-LNP induces antitumor effects in nonhepatic tumor models.

To evaluate whether DNA-LNP can induce antitumor responses in nonhepatic tumors, we evaluated its effect in mouse models of acute myeloid leukemia (AML), s.c. melanoma, and melanoma lung metastasis.

**Figure 3:**
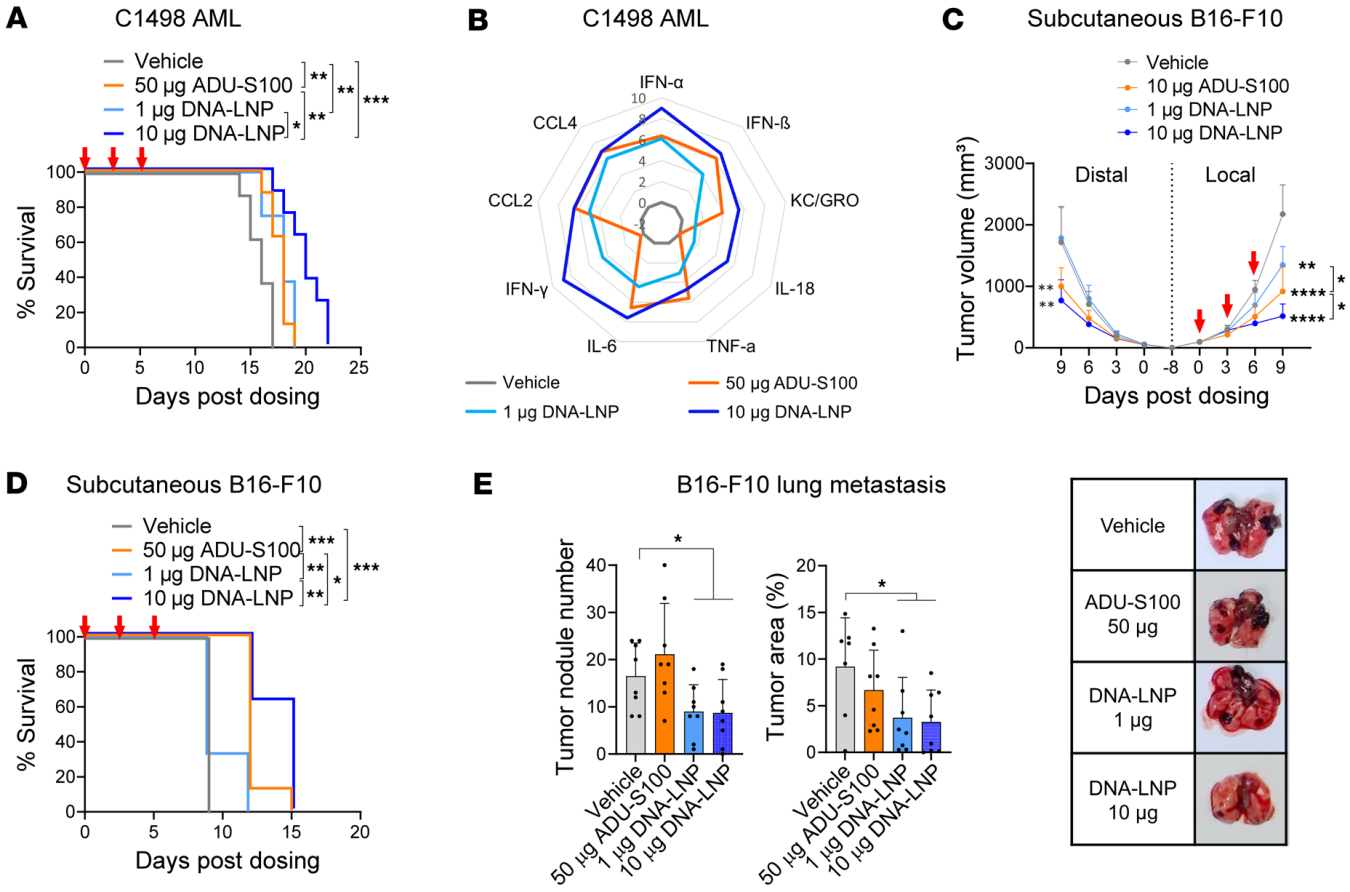
DNA-LNP induces an antitumor effect in nonhepatic tumor models. (**A** and **B**) C1498 AML tumor-bearing mice (*n* = 8) were i.v. dosed with DNA-LNP or ADU-S100 at days 0, 3, and 6. (**A**) Survival curves. (**B**) Serum cytokine levels 4 hours after the first dose. Average values are represented as log_2_ fold-increase over vehicle. (**C** and **D**) B16-F10 tumors were inoculated to both sides of mice (*n* = 8) and intratumorally dosed with treatments at 1 tumor site (local) at days 0, 3, and 6. (**C**) Tumor growth curves of melanoma-bearing mice. *P* value indicates comparison to the vehicle-treated group at day 9 unless otherwise indicated. (**D**) Survival curves. (**E**) Tumor nodule numbers on the lung surface, tumor area from lung sections, and representative images of the lungs from the B16-F10 lung metastasis model at day 13. Mice (*n* = 8) were i.v. dosed at days 0, 3, and 6. Data shown as mean ± SD. Survival data were analyzed by log-rank (Mantel-Cox) tests, and other data were analyzed by 2-tailed Student’s *t* test. *P < 0.05, **P < 0.01, ***P < 0.001, and ****P < 0.0001.

We first established an AML model by i.v. inoculating C1498 cells into mice. Three days after inoculation, the mice were i.v. dosed with either vehicle (20% glucose-PBS), DNA-LNP, or ADU-S100, a clinically tested STING agonist used as a control. DNA-LNP administration prolonged the survival of C1498 AML-bearing mice in a dose-dependent manner, demonstrating that 10 μg of DNA-LNP was more potent than 50 μg of ADU-S100 (Figure 3A). Importantly, DNA-LNP and ADU-S100 resulted in distinct serum cytokine profiles, with both inducing type I IFNs (IFN-α, IFN-β), cytokines (TNF-α, IL-6), and chemokines (CCL2, CCL4, KC/GRO-α), but only DNA-LNP induced IL-18 and IFN-γ (Figure 3B). IL-18, which was reported to be induced by the AIM2-inflammasome pathway after DNA-LNP dosing (21), is known to enhance production of IFN-γ, a critical factor for antitumor T cell responses (31). Extending the DNA-LNP regimen to 6 doses administered every 3 days did not result in an additional survival benefit compared with the 3-dose schedule (Supplemental Figure 2D). Reducing the frequency of DNA-LNP dosing to 3 doses 6 days apart or 2 doses 12 days apart maintained the antitumor effect observed with the standard schedule of 3 doses administered 3 days apart (Supplemental Figure 3A).

Next, we evaluated the antitumor effect of DNA-LNP in the bilateral s.c. B16-F10 mouse melanoma model. B16-F10 cells were inoculated into both flanks, with one flank (distal) left untreated and the other flank (local) then dosed intratumorally with DNA-LNP or ADU-S100 on a schedule of 3 doses given 3 days apart, a dosing schedule used in previous studies evaluating STING agonists (32, 33). DNA-LNP demonstrated a dose-dependent effect on tumor size of the local flank, and the higher dose of DNA-LNP also reduced the size of the distal tumor (Figure 3C). Consistently, DNA-LNP extended the survival of melanoma-bearing animals in a dose-dependent manner (Figure 3D). Furthermore, a 10 μg dose of DNA-LNP showed a more effective antitumor effect than 10 μg of ADU-S100 (Figure 3, C and D). Reducing the frequency of DNA-LNP dosing to 2 doses 6 days apart still effectively reduced B16-F10 tumor size and prolonged animal survival (Supplemental Figure 3, B and C). Intratumorally dosing DNA-LNP induced detectable levels of cytokines in the serum (Supplemental Figure 4), but cytokine induction was substantially lower compared with i.v. dosing (Figure 3B). For example, the level of serum IFN-α induced by intratumoral administration of 10 μg of DNA-LNP was 13 times lower compared with i.v. administration in the AML model.

Lastly, we assessed the effect of DNA-LNP in a B16-F10 melanoma lung metastasis model. Mice were inoculated with B16-F10 cells via the tail vein, and metastases were allowed to develop in the lungs over a period of 5 days before i.v. administering DNA-LNP or ADU-S100. To evaluate the antitumor effect, lung tissues were isolated 13 days after treatment, and tumor nodules and tumor area were analyzed from the surface and sections of the lung tissue, respectively. Although 50 μg of ADU-S100 did not induce an antitumor effect in the melanoma lung metastasis model, even the low dose (1 μg) of DNA-LNP reduced the number of lung tumor nodules and tumor area (Figure 3E and Supplemental Figure 5).

Overall, the strong antitumor effect induced by both local and systemic dosing of DNA-LNP in multiple nonhepatic tumor models (AML, s.c. melanoma, and melanoma lung metastasis) suggests a generalized therapeutic effectiveness of DNA-LNP that is not limited to a particular tumor type or route of administration.

### DNA delivered by LNP mediates the antitumor effect in nonhepatic tumor models.

To investigate key determinants of DNA-LNP–induced antitumor activity in nonhepatic tumor models, we prepared DNA-LNP formulations with an alternative ionizable lipid (DNA-LNP2) or encapsulating a shorter (1.3 kb vs. 5.1 kb) noncoding DNA with a distinct sequence (DNA2-LNP), as well as LNPs without payload (empty LNPs).

**Figure 4:**
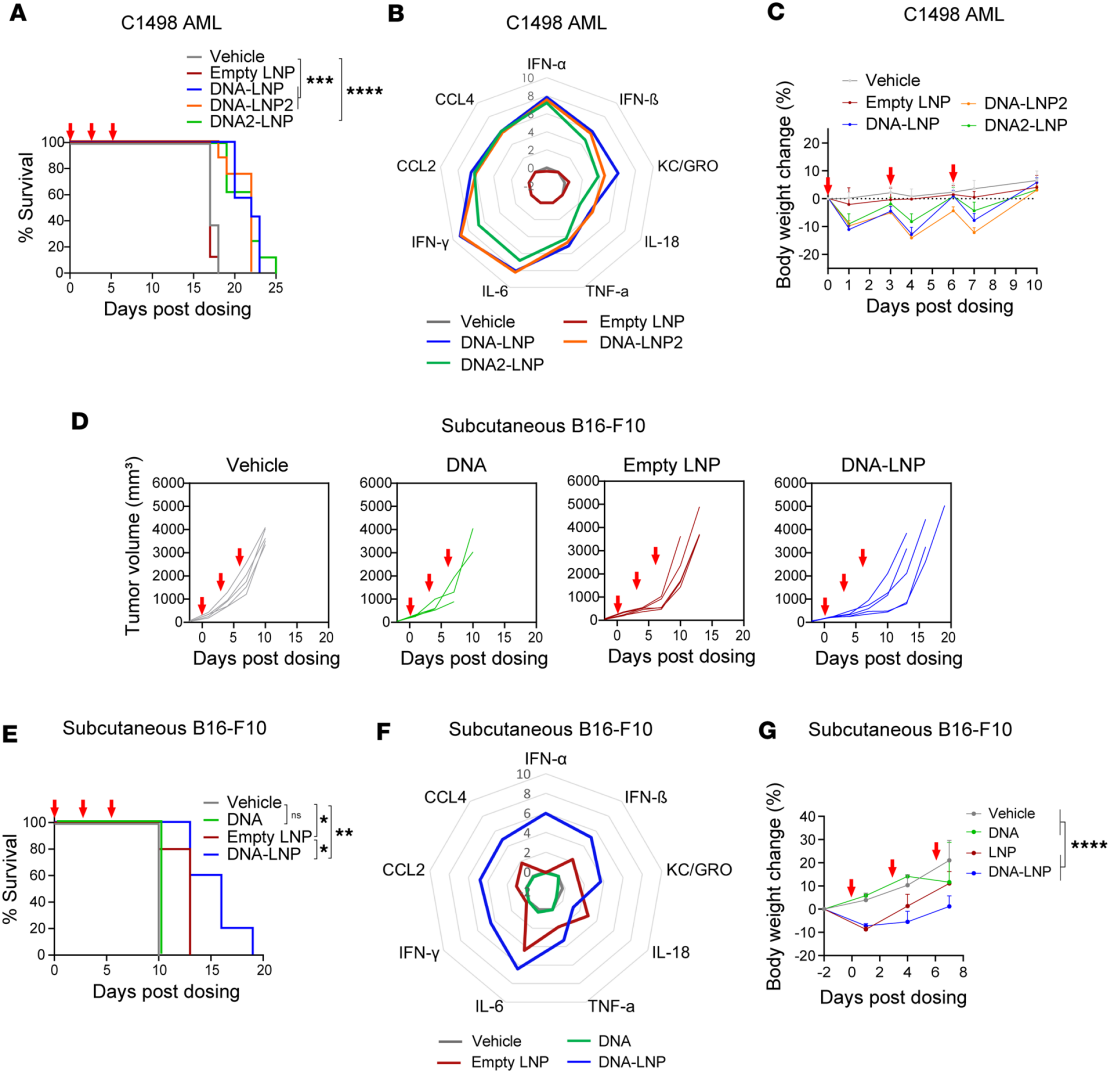
DNA delivered by LNP mediates the antitumor effect in nonhepatic tumor models. (**A**–**C**) C1498 AML-bearing mice (*n* = 8) were i.v. dosed with 5 μg of DNA-LNPs or an equivalent dose of empty LNP at days 0, 3, and 6. (**A**) Survival curves. (**B**) Serum cytokine levels 4 hours after dosing. Average values are represented as log_2_ fold-increase over vehicle. (**C**) Body weight loss measured over time. Data shown as mean ± SD. (**D**–**G**) Mice (*n* = 4~5) with B16-F10 tumor at 1 side were intratumorally dosed with 10 μg of DNA, DNA-LNP, or an equivalent dose of empty LNP at days 0, 3, and 6. (**D**) Tumor growth curves. (**E**) Survival curves. (**F**) Serum cytokine levels 4 hours after the first dosing. Average values are represented as log_2_ fold-increase over vehicle. (**G**) Body weight loss. Statistical analysis was done at 1 day after dosing data using 1-way ANOVA with Tukey’s multiple-comparison test. Survival data were analyzed by log-rank (Mantel-Cox) tests. *P < 0.05, **P < 0.01, ***P < 0.001, ****P < 0.0001, and ns (not significant).

We evaluated the effect of these LNPs in the AML model after i.v. administration. Empty LNPs did not induce antitumor effects, serum cytokines, or transient body weight loss (Figure 4, A–C), indicating that the immunostimulatory and antitumor effects are specifically mediated by LNP-mediated delivery of DNA. DNA-LNP, DNA-LNP2, and DNA2-LNP showed similar antitumor effect, cytokine profile, and transient body weight loss (Figure 4, A–C). DNA2-LNP showed a milder cytokine profile compared with DNA-LNP (Figure 4B), similar to what we observed in the HCC model (Figure 2J). These results demonstrate that the immunostimulatory and antitumor effects of DNA-LNP are not necessarily specific to a particular DNA payload or ionizable lipid.

We then evaluated the role of DNA-LNP components in the s.c. B16-F10 model after local administration to the tumor. DNA alone without LNP did not induce antitumor effects, serum cytokines, or body weight loss that was induced by DNA-LNP (Figure 4, D–G), demonstrating a requirement for LNP-mediated DNA delivery. In contrast to i.v. dosing of empty LNPs, local administration of empty LNPs in the melanoma model induced detectable levels of antitumor effects, cytokines, and body weight loss (Figure 4, D–G). However, the antitumor effect from locally dosed empty LNPs was substantially lower than DNA-LNPs (Figure 4E), indicating that DNA delivery is critical for the full antitumor effect of DNA-LNP.

Altogether, these results demonstrate that the immune responses and antitumor effects induced by DNA-LNP require LNP-mediated DNA delivery and that the DNA payload or ionizable lipid can be modified while maintaining the antitumor effect.

### DNA-LNP induces CD8^+^ T cell infiltration of tumor sites and CD8^+^ T cell–mediated antitumor effect.

**Figure 5:**
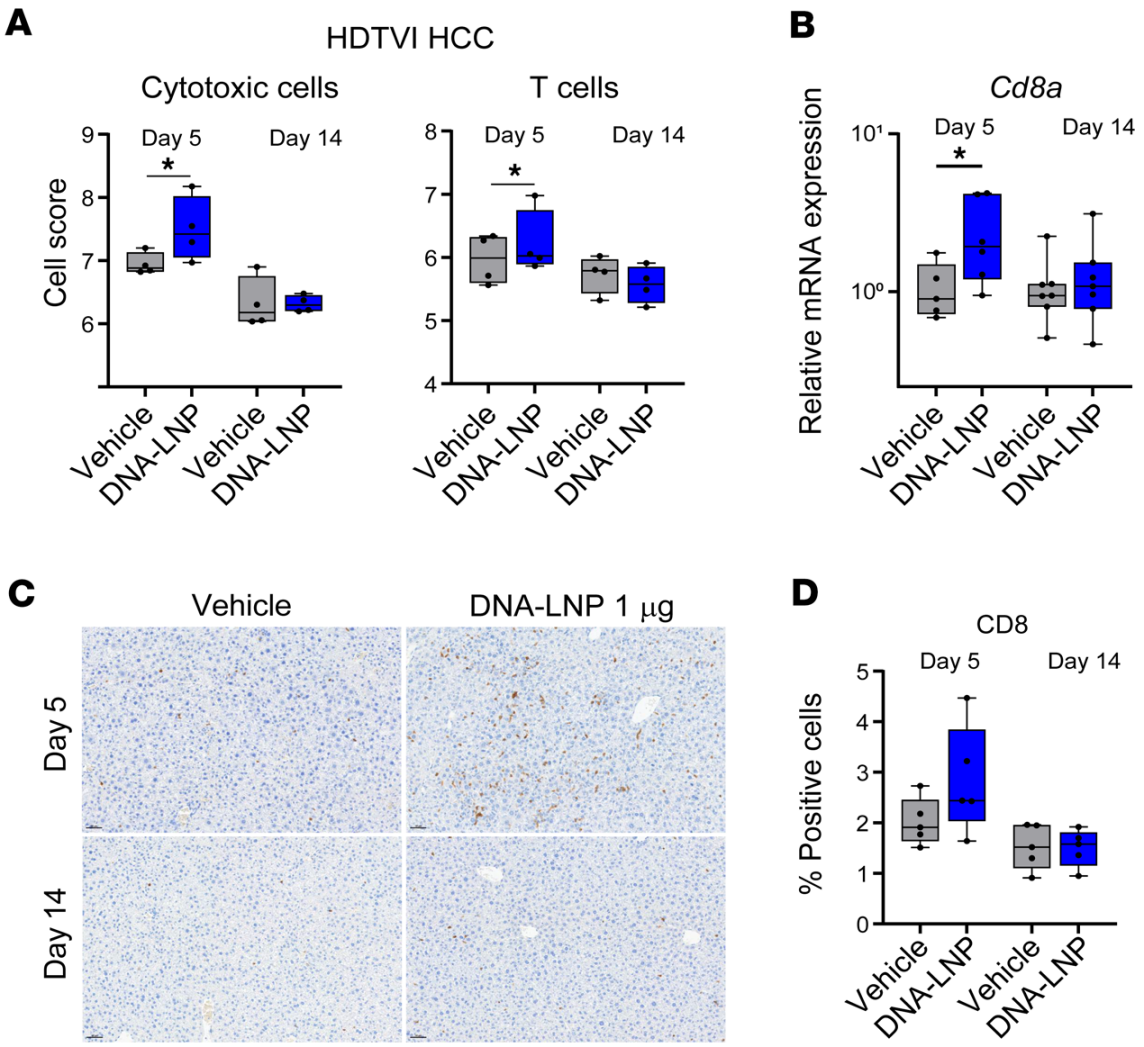
DNA-LNP induces CD8^+^ T cell infiltration of the liver in HCC mouse model. HDTVI-induced HCC-bearing mice received a single i.v. dose of 1 μg of DNA-LNP 3 weeks after HCC induction. Livers were harvested and analyzed at days 5 and 14 after treatment. (**A**) Cell-type profiling by NanoString. (**B**) qRT-PCR analysis of CD8a. (**C**) Representative images (20×) and (**D**) quantification of IHC analysis of CD8^+^ cells from liver sections. Min-to-max whiskers are shown in the box and whiskers plot. Data were analyzed by 1-way ANOVA with Tukey’s multiple-comparison test. *P < 0.05.

To investigate the mechanisms underlying the antitumor effects of DNA-LNP, we performed cell-type profiling (NanoString Technologies) to analyze changes after treatment in immune cell infiltration in the livers of mice with genetically induced HCC. Although this analysis was conducted on bulk liver tissue, this method leverages curated multi-gene expression signatures to estimate the relative abundance of specific immune cell populations. At day 5 after treatment, we observed an enrichment of cytotoxic cells (defined by the expression of *Prf1*, *Klrb1*, *Klrd1*, *Klrk1*, *Nkg7*, *Gzma*, *Gzmb*, and *Ctsw*) and T cells (defined by the expression of *Cd3d*, *Cd3e*, *Cd3g*, *Cd6*, *Sh2d1a*, and *Trat1*) in the liver, which was resolved by day 14 (Figure 5A). No statistical differences between treated and control mice were observed in exhausted CD8^+^ T cells (defined by the expression of *Cd244*, *Eomes*, *Lag3*, and *Ptger4*) or other immune cell populations, including NK cells (Supplemental Figure 6). These findings were further corroborated by RT-PCR, which demonstrated an increase in *Cd8a* expression in the liver at day 5 but not at day 14 after treatment (Figure 5B). Additionally, although not statistically significant, IHC analysis of liver sections showed a trend of increased CD8^+^ cells (Figure 5, C and D).

**Figure 6:**
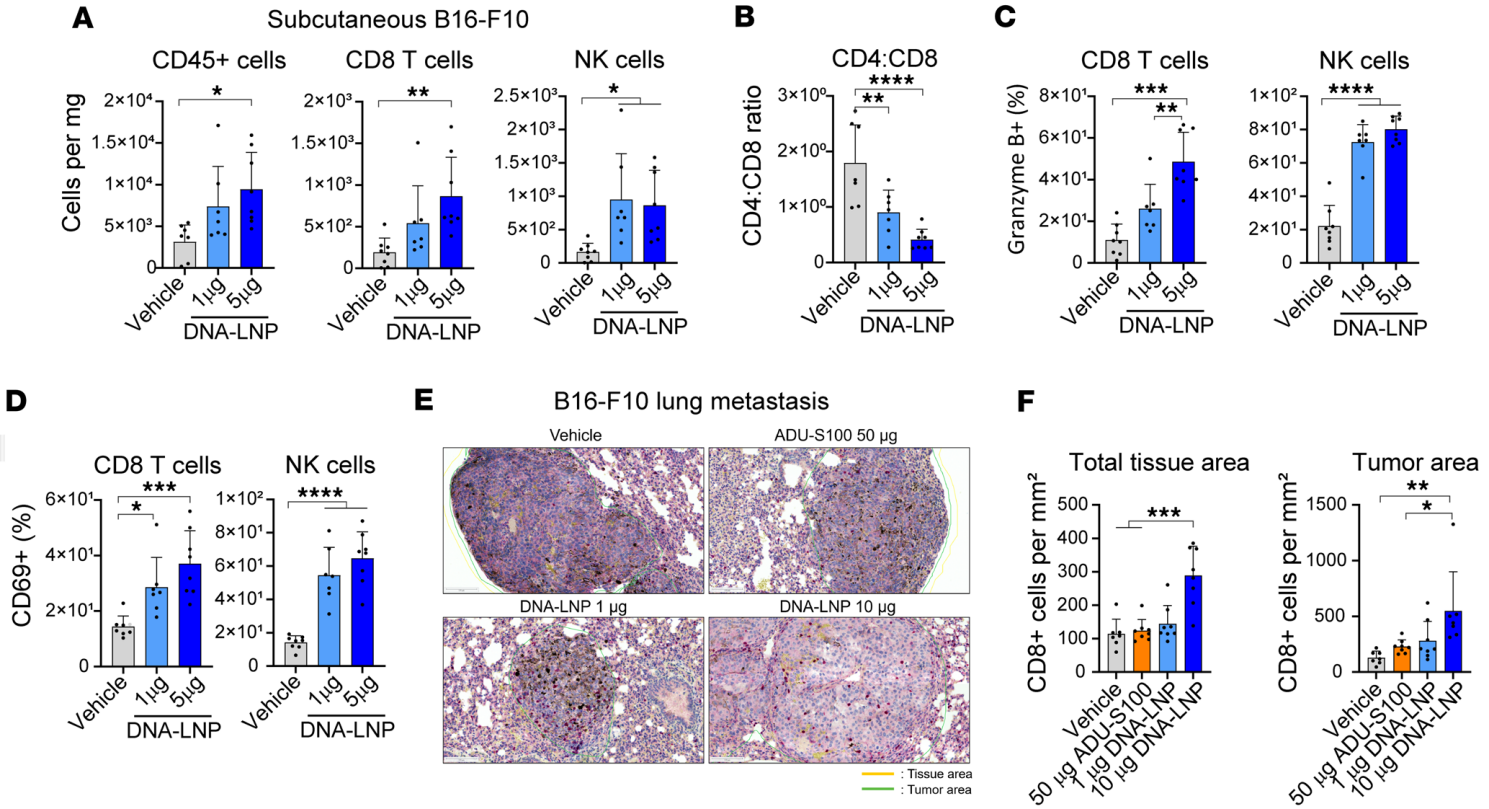
DNA-LNP induces CD8^+^ T cell infiltration of tumor sites and immune cell activation in nonhepatic tumor models. (**A**–**D**) B16-F10 tumor-bearing mice (s.c.) were intratumorally dosed with DNA-LNP at days 0 and 3. Tumors were collected at day 7 and analyzed using flow cytometry. (**A**) Numbers of indicated immune cells per milligram of tumor tissues. (**B**) CD4/CD8 ratio. (**C**) Percentage of Granzyme B^+^ cells and (**D**) CD69^+^ cells from CD8^+^ T cells or NK cells. (**E** and **F**) Mice with B16-F10 lung metastasis were i.v. dosed at days 0, 3, and 6, and lung sections were analyzed at day 13 by CD8 IHC. (**E**) Representative images of lung sections and (**F**) quantification of CD8^+^ cells. Min-to-max whiskers are shown in the box and whiskers plot. Data shown as mean ± SD. Data were analyzed by 1-way ANOVA with Tukey’s multiple-comparison test. *P < 0.05, **P < 0.01, ***P < 0.001, and ****P < 0.0001.

In the s.c. B16-F10 melanoma model, intratumoral dosing of DNA-LNP increased the number of total immune cells, CD8^+^ T cells, and NK cells within the tumor in a dose-dependent manner (Figure 6A and Supplemental Figure 7A). The ratio of CD4^+^ to CD8^+^ T cells was reduced by DNA-LNP, suggesting an accumulation of cytotoxic T cells in the tumor (Figure 6B). Furthermore, DNA-LNP upregulated Granzyme B and CD69 in tumor-infiltrating CD8^+^ T cells and NK cells, indicating activation of these immune cells (Figure 6, C and D, and Supplemental Figure 7, B and C). Moreover, in the B16-F10 lung metastasis model, i.v. dosing of DNA-LNPs increased the number of CD8^+^ cells in both total and tumor tissue areas, whereas ADU-S100 did not show a difference (Figure 6, E and F).

**Figure 7:**
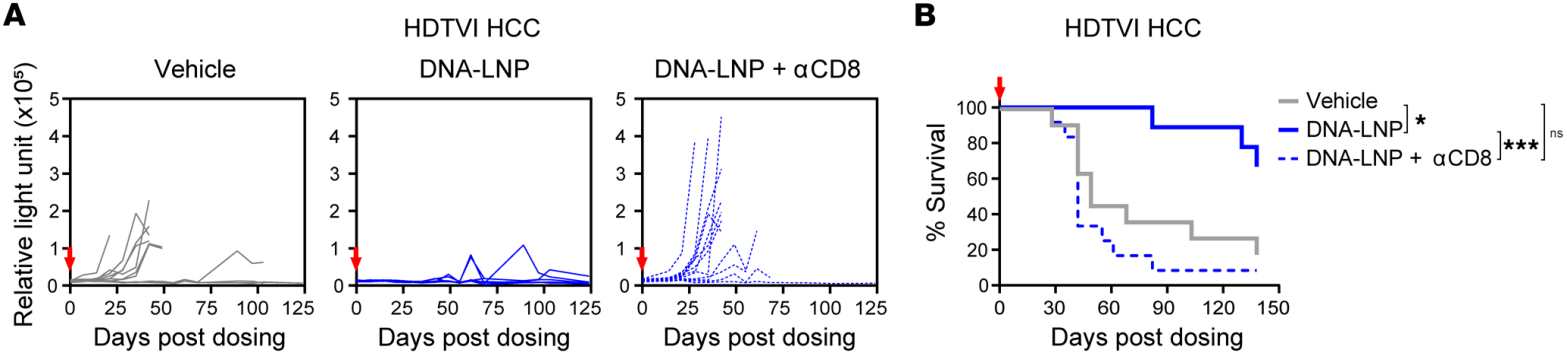
DNA-LNP induces CD8^+^ T cell–mediated antitumor effect in HCC mouse model. HDTVI-induced HCC-bearing mice (*n* = 11) received a single i.v. dose of 1 μg of DNA-LNP 3 weeks after HCC induction. Mice received 200 μg of anti-CD8 or anti-NK1.1 (i.p.) at days –2, 1, 4, and 7 to deplete CD8^+^ T cells or NK cells, respectively. (**A**) Tumor growth indicated by serum GLuc activity. (**B**) Survival curves. Survival data were analyzed by log-rank (Mantel-Cox) tests. *P < 0.05, ***P < 0.001, and ns (not significant).

**Figure 8:**
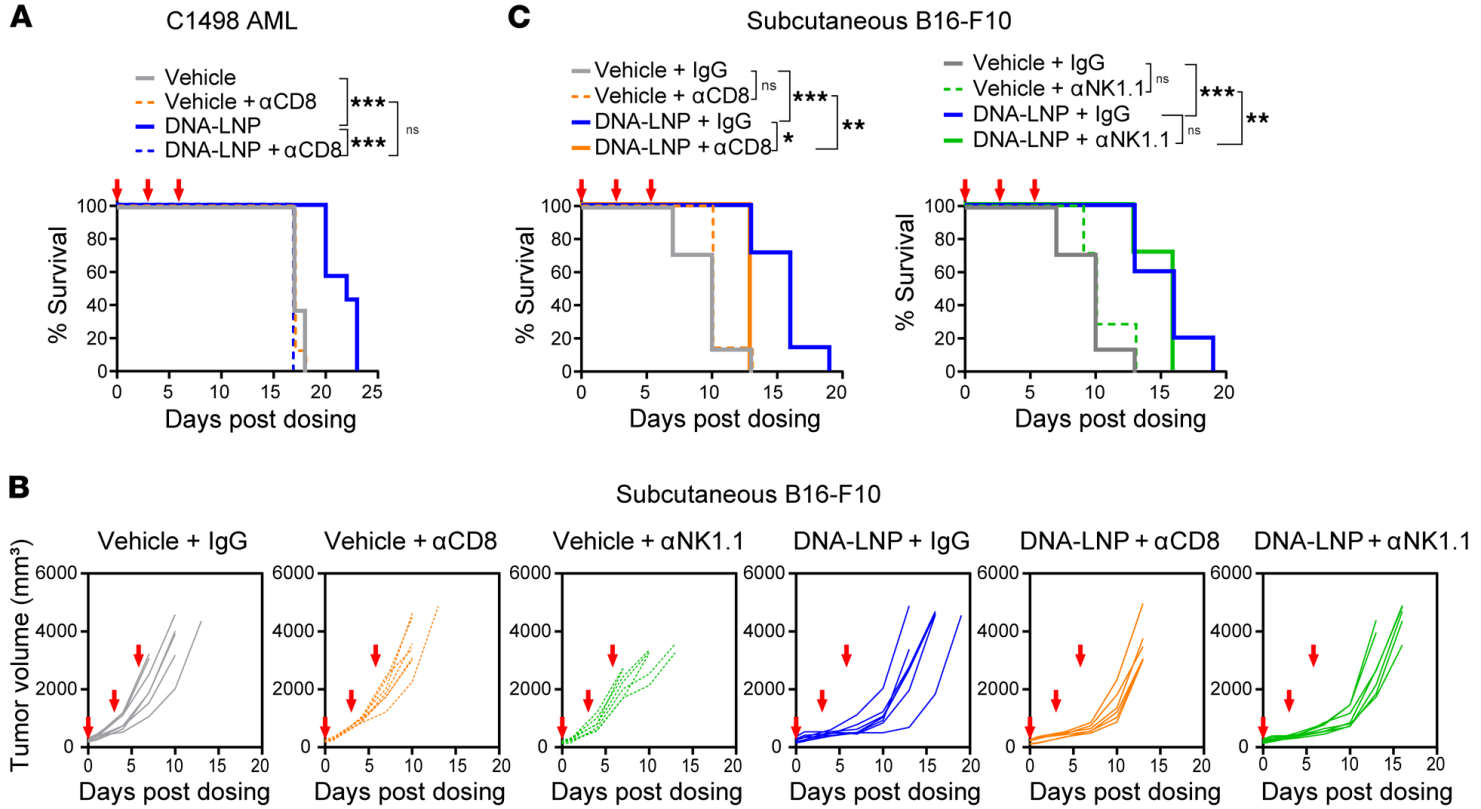
DNA-LNP induces CD8^+^ T cell–mediated antitumor effect in nonhepatic tumor models. Mouse tumor models received 200 μg of anti-CD8 or anti-NK1.1 (i.p.) at day –2, 1, 4, and 7 to deplete CD8^+^ T cells or NK cells, respectively. (**A**) Survival curves of C1498 AML tumor-bearing mice (*n* = 8) after i.v. administration of 5 μg of DNA-LNP at days 0, 3, and 6. (**B**) Tumor growth curves and (**C**) survival curves of B16-F10 tumor-bearing mice (*n* = 7) after intratumoral administration of 10 μg of DNA-LNP at days 0, 3, and 6. Survival data were analyzed by log-rank (Mantel-Cox) tests. *P < 0.05, **P < 0.01, ***P < 0.001, and ns (not significant).

To determine whether the increased CD8^+^ T cell infiltration directly contributes to the antitumor response induced by DNA-LNP, we depleted CD8^+^ T cells prior to DNA-LNP treatment. In the genetically induced HCC mouse model, the antitumor effect was completely abolished in the absence of CD8^+^ T cells, as evidenced by the loss of antitumor efficacy (Figure 7A) and decreased animal survival (Figure 7B). Similarly, the prolonged survival effect by DNA-LNP in the C1498 AML model was not observed after depleting CD8^+^ T cells (Figure 8A). In the s.c. B16-F10 melanoma model, CD8^+^ T cell depletion reduced but did not completely abolish the antitumor efficacy of DNA-LNP, indicated by reduced tumor growth control (Figure 8B) and shortened animal survival (Figure 8C). This result suggests that although CD8^+^ T cells are key mediators of DNA-LNP–induced antitumor activity in this model, additional mechanisms may also contribute. NK cells were recruited and activated after DNA-LNP administration in this model (Figure 6, A, C, and D), but their depletion did not result in a substantial loss of antitumor effect (Figure 8, B and C). Further studies will be required to elucidate the additional immune components that mediate the antitumor effect. Together, these data indicate that DNA-LNPs can increase the number and the activity of tumor-infiltrating lymphocytes across multiple tumor models, effectively transforming cold tumors into hot tumors and inducing CD8^+^ T cell–mediated antitumor effects.

### DNA-LNP enhances the antitumor effect of anti–PD-L1.

**Figure 9:**
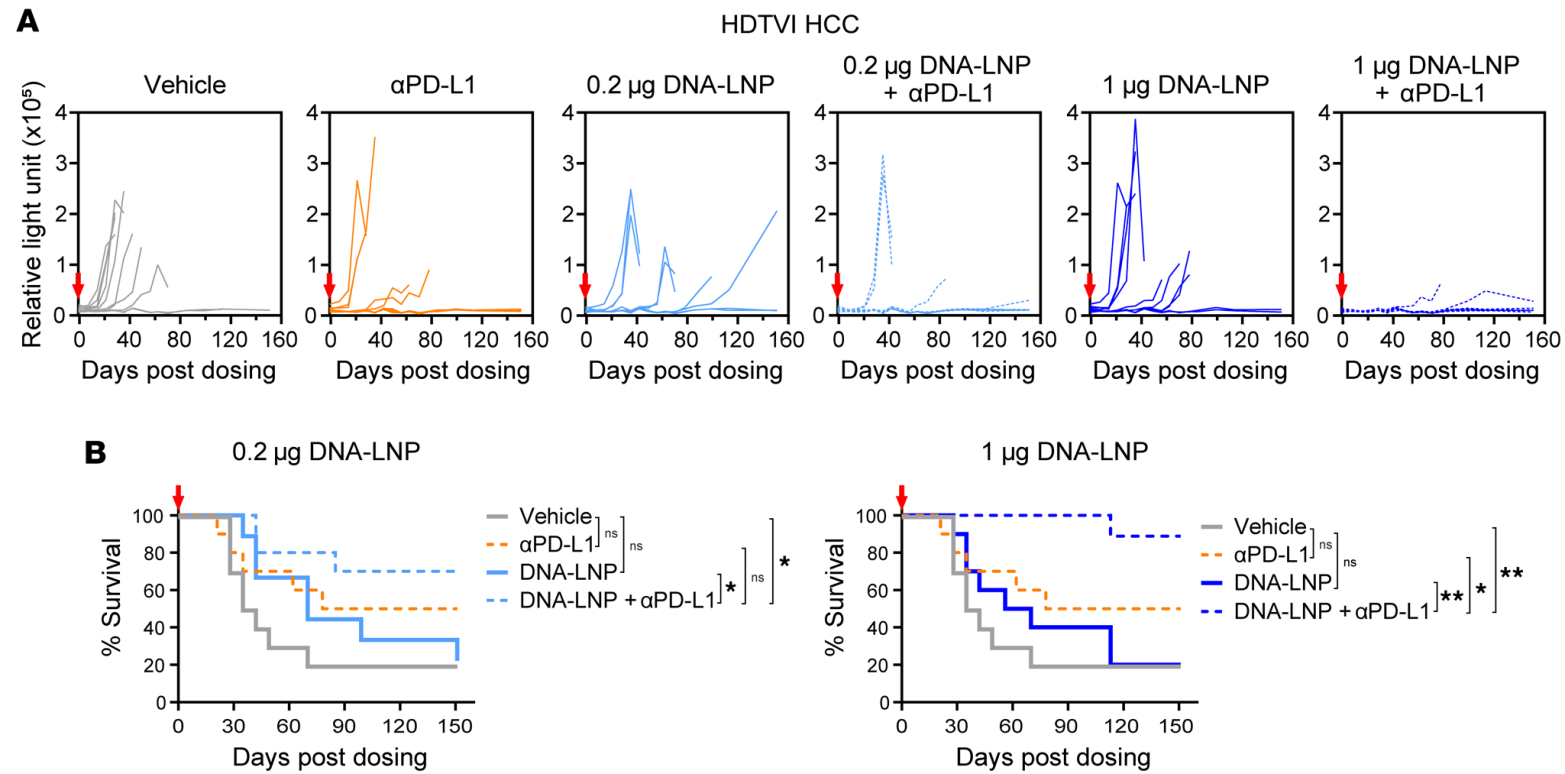
DNA-LNP and anti–PD-L1 show synergistic antitumor effects in HCC mouse model. HDTVI-induced HCC models (*n* = 11) were i.v. dosed with the indicated doses of DNA-LNP 4 weeks after HDTVI to observe a suboptimal antitumor effect. At days 1, 5, 8, and 11, 200 μg of anti–PD-L1 was i.p. dosed. (**A**) Tumor growth indicated by serum GLuc activity. (**B**) Survival curves of HCC-bearing mice. Survival data were analyzed by log-rank (Mantel-Cox) tests. *P < 0.05, **P < 0.01, and ns (not significant).

ICI therapy has limited effectiveness in some patients. Combination therapies are actively being evaluated in clinical trials to amplify therapeutic effects and benefit patients who do not respond to ICIs. To boost the antitumoral effect of low-dose DNA-LNP, we tested DNA-LNP in combination with an ICI, anti–PD-L1. First, we combined the administration of DNA-LNP with anti–PD-L1 antibodies in the genetically induced HCC mouse model. To test the therapy under a more demanding disease setting, treatment start was extended from 3 to 4 weeks after tumor induction. Under these conditions, the monotherapy treatments tested, anti–PD-L1 or DNA-LNP at doses of either 0.2 or 1 μg, resulted in a mild antitumor effect, but failed to control long-term tumor progression (Figure 9A) or to substantially extend mouse survival compared with vehicle-treated mice (Figure 9B). Remarkably, a synergistic effect of the combination treatment was observed for both DNA-LNP doses. At the higher dose (1 μg), 90% of treated mice were disease-free at the study’s endpoint, and at the lower dose (0.2 μg), 70% of animals remained disease-free by day 151 after treatment (Figure 9B).

**Figure 10:**
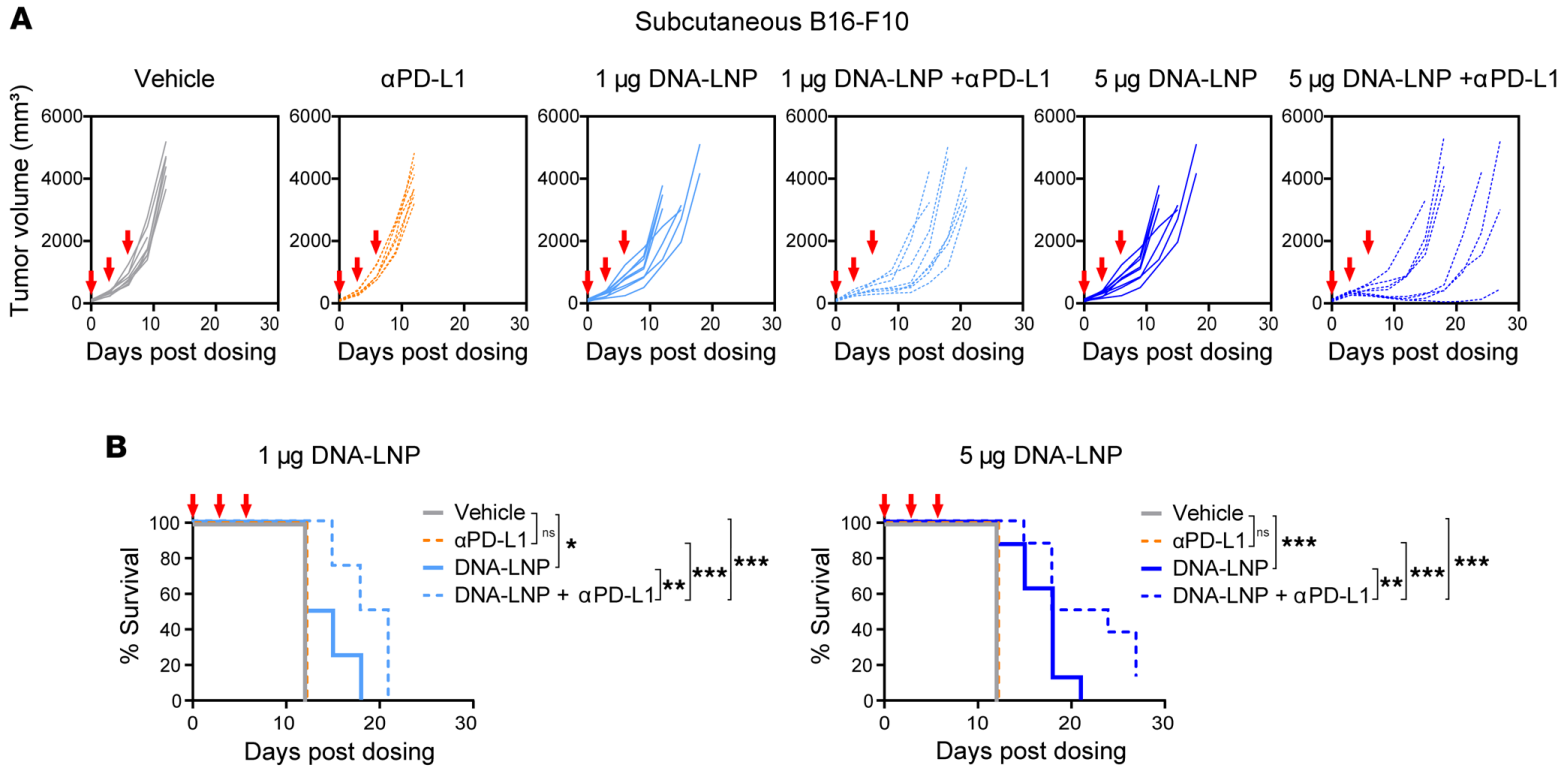
DNA-LNP and anti–PD-L1 show synergistic antitumor effects in anti–PD-L1-resistant melanoma model. B16-F10 tumor-bearing mice (*n* = 8) were i.p. dosed with 250 μg of anti–PD-L1 and intratumorally dosed with indicated doses of DNA-LNP at days 0, 3, and 6. (**A**) Tumor growth curves. (**B**) Survival curves. Survival data were analyzed by log-rank (Mantel-Cox) tests. *P < 0.05, **P < 0.01, ***P < 0.001, and ns (not significant).

We next evaluated the combination of DNA-LNP with anti–PD-L1 in the s.c. B16-F10 model, which is resistant to anti–PD-L1 treatment. As expected, anti–PD-L1 alone did not have an antitumor effect (Figure 10, A and B). However, when combined with DNA-LNP, anti–PD-L1 enhanced the therapeutic effect, resulting in better control of tumor progression (Figure 10A) and prolonged survival (Figure 10B).

Notably, combining DNA-LNP with anti–PD-L1 antibodies allowed for a reduction in the effective DNA-LNP dose. In both models, low-dose DNA-LNP combined with anti–PD-L1 antibodies achieved a therapeutic effect equivalent to that of a 5-fold higher dose of DNA-LNP alone. Furthermore, the combination with anti–PD-L1 did not exacerbate body weight loss observed with DNA-LNP treatment in any of the tumor models (Supplemental Figure 8, A and B). Therefore, the CD8^+^ T cell–dependent antitumor activity of DNA-LNPs can be successfully combined with anti–PD-L1 to further boost responses in both models, HCC responsive to anti–PD-L1 and melanoma resistant to anti–PD-L1, suggesting potentially broad application to tumors with varying levels of ICI responsiveness.

## Discussion

Immunostimulatory drugs can induce antitumor immune responses and potentiate the effect of ICI treatment. However, early clinical trials have underscored the need for further enhancement of these immune responses. In this study, we evaluated the capacity of DNA-LNPs, which activate STING and the AIM2 inflammasome (21), to induce effective antitumor responses. Our results show that noncoding DNA-LNP induces CD8^+^ T cell–dependent antitumor responses in multiple tumor models. In addition, combining DNA-LNPs with anti–PD-L1 therapy results in a synergistic antitumor effect in both models, HCC responsive to anti–PD-L1 and melanoma resistant to anti–PD-L1. These data highlight the potential of DNA-LNPs as an immunotherapy alone or in combination with ICIs.

We previously demonstrated that DNA-LNPs induce multiple immune mediators, including type I IFNs, key drivers of antitumor immunity (21). Blockade of type I IFN signaling markedly reduced IFN-γ levels (21), suggesting that type I IFN induction may be a major contributor to the antitumor effects of DNA-LNPs observed here. Targeted inhibition with anti-IFNAR antibodies or JAK inhibitors in tumor-bearing mice could formally validate this pathway. The CD8^+^ T cell–dependent antitumor effect further suggests expansion of antigen-specific T cells. Although local DNA-LNP dosing can lead to limited systemic exposure and activation of distal immune cells, the LNPs do not carry tumor antigens; thus, tumor antigen presentation is expected to occur predominantly in tumor-draining lymph nodes or tertiary lymphoid structures, as seen with local adjuvants and mRNA-LNP vaccines (34–37). Detailed characterization of these compartments will identify where antigen presentation occurs and will assess the quantity and functional properties of the responding T cells.

In our study, a DNA payload was essential for the full antitumor activity of DNA-LNPs, as empty LNPs exhibited minimal immunostimulatory effects, particularly with i.v. dosing. Although empty LNPs or LNPs carrying nonimmunogenic cargo can elicit immune responses primarily via their ionizable lipid components, this lipid-driven immunogenicity is substantially weaker than the robust inflammatory responses triggered by cytosolic DNA (38). This is consistent with prior reports showing that high i.v. doses of mRNA-LNPs are required to elicit detectable serum cytokines (39). In contrast, intratumoral administration of empty LNPs produced measurable serum cytokines and modest antitumor effects in the s.c. melanoma model. This observation aligns with previous vaccination studies showing that intramuscular delivery of empty LNPs or chemically modified mRNA-LNPs can provoke inflammatory responses even at relatively low doses (40, 41). Local administration likely concentrates LNPs within the tumor microenvironment, facilitating uptake by resident immune cells and contributing to these minor antitumor effects. Although the inflammatory response induced by empty LNPs was modest, human immune systems are generally more sensitive to LNPs than those of murine models, partly due to differences in IL-1 pathway regulation (42). Further research should define how LNP formulation influences immune responses and the antitumor effects of DNA-LNPs in humans. Moreover, the therapeutic potential of DNA-LNPs may be enhanced by strategically optimizing LNP immunogenicity in combination with immunostimulatory DNA payloads.

We also evaluated the impact of DNA payload characteristics on antitumor efficacy. In this study, 2 distinct DNA constructs were tested — one 5.1 kb with no CpG motifs and another 1.3 kb with 16 CpG motifs — both of which demonstrated antitumor activity across multiple models. These findings suggest that both the sequence and length of the DNA payload can be modified without compromising therapeutic efficacy. We previously investigated the role of TLR9, an endosomal receptor for unmethylated CpG (43), in mediating DNA-LNP immunogenicity. We demonstrated that CpG-containing and CpG-depleted DNA-LNPs induced similar innate immune responses in both WT and TLR9-KO mice (21), indicating that CpG motifs are not major contributors to DNA-LNP immunogenicity. Instead, the cGAS/STING and AIM2 pathways emerged as the key mediator of these responses. DNA length may influence the magnitude of the response, as cGAS and AIM2 recognize dsDNA in a sequence-independent manner but display length-dependent activity (44–48). Whether specific sequences or lengths of DNA can further enhance antitumor efficacy remains to be explored to fine-tune immune activation.

Since the length or sequence of DNA payload can be modified, DNA can be engineered to encode transgenes to further improve therapeutic efficacy. Antitumor transgenes are already employed in other contexts. For example, Talimogene laherparepvec, an oncolytic virus approved for metastatic melanoma, encodes GM-CSF to enhance immune responses (49). Subsequent studies demonstrated improved preclinical efficacy when adenoviruses and oncolytic viruses encoded suicide genes or membrane-bound or secreted immunostimulatory molecules (50–52). Additionally, a recent report showed antitumor activity after intratumoral LNP co-delivery of DNA encoding OX40 ligand and siRNA targeting indoleamine 2,3 dioxygenase in a mouse s.c. melanoma model (18).

LNPs can encapsulate other therapeutic payloads alongside DNA to provide enhanced antitumor effects. Multiple siRNA-LNPs have been evaluated to reduce oncogene or immunosuppressive gene expression (53). In addition, mRNA-LNPs expressing therapeutic antibodies or immunostimulatory factors are now in clinical trials (54). Other approaches encapsulate chemotherapy drugs in LNPs to minimize exposure to healthy tissues while increasing drug half-life (55). Similarly, STING agonists encapsulated in LNP enhanced antitumor effects in glioblastoma and lung metastasis models (56, 57). Given the complex mechanisms of tumor progression and immune evasion, engaging multiple pathways by co-encapsulating these drugs into DNA-LNPs or formulating them into distinct LNPs may provide improved therapeutic benefits.

LNP delivery can be optimized to target tumors while minimizing exposure to other organs. In this study, an LNP with high liver tropism produced antitumor effects after local or systemic dosing. Although advantageous for HCC, liver-tropic LNPs may be less suitable for nonhepatic tumors because of preferential liver accumulation. Enhancing tumor-specific delivery through LNP modification could improve local immune responses and limit potential systemic side effects. Optimization of LNP components and compositions has been shown to de-target LNPs from the liver and promote accumulation in specific organs (54). In addition, ligands such as small sugars, peptides, and antibodies can actively target LNPs to specific cells (58). These advances can be applied to improve LNP delivery to specific cancerous organs or cells and thereby enhance efficacy and tolerability.

Despite the recent inclusion of PD-1/PD-L1–blocking antibodies with CTLA-4 or VEGF-A blockade as first-line therapy for advanced HCC — following superior activity over sorafenib — only 17%–36% of patients respond to these therapies (59–61). Similarly, although anti–PD-L1 and other ICI therapies are approved for melanoma, most patients fail to respond or later develop resistance (62). Here, we show that DNA-LNP can enhance tumor sensitivity to anti–PD-L1 treatment by promoting CD8^+^ T cell recruitment to the tumor microenvironment, improving therapeutic efficacy in both HCC and melanoma models. Notably, PD-1 expression has been used to identify tumor-reactive lymphocytes in immunotherapy settings (63, 64), suggesting that the combination therapy may augment the activity of tumor-specific T cells. Although synergy with anti–PD-L1 may suggest PD-1^+^ CD8^+^ T cells, our cell-type profiling in HCC showed increased infiltration of T cell and cytotoxic compartments without clear evidence of exhaustion. Single-cell analyses of specific exhaustion markers will be necessary to clarify phenotypic changes and dynamics in the CD8^+^ T cell compartment after treatment.

Achieving robust antitumor efficacy while minimizing systemic adverse effects remains a fundamental challenge in oncology (65). In our previous study, high DNA-LNP doses (25–50 μg) were required for productive hepatocyte transgene expression in vivo — a process that relies on relatively inefficient nuclear delivery — and were accompanied by type I IFN–dependent morbidity (21). In contrast, the current study uses substantially lower doses (0.2–10 μg) to elicit antitumor effects without overt morbidity as the noncoding DNA activates STING in the cytosol rather than driving transgene expression, permitting much lower DNA-LNP doses. Nonetheless, transient, dose-dependent body weight loss was observed above 1 μg: mice given 10 μg recovered within a few days without further distress, and no weight loss was observed at 0.2 μg. Systemic cytokines rose transiently but returned to baseline within 24 hours, consistent with a reversible, immune-related response. Clinically, mild to moderate treatment-related events, including transient weight loss, are common with immunotherapies and are generally manageable (59, 66, 67). In patients with HCC, immune-related adverse events have been associated with improved outcomes, suggesting that controlled immune activation may accompany effective antitumor responses (66). Although the clinical relevance of these transient effects requires further investigation, our data support combination strategies — such as DNA-LNPs with anti–PD-L1 — that allow dose reduction without exacerbating adverse effects. Comprehensive toxicity studies will be essential prior to clinical translation.

Overall, our findings underscore the potential of DNA-LNP as a versatile platform for cancer immunotherapy. By efficiently delivering DNA to activate innate immune pathways and enhance adaptive immune responses, DNA-LNP can induce potent antitumor effects either alone or in combination with ICIs. Future approaches may further improve standalone efficacy by incorporating therapeutic transgenes, optimizing LNP delivery and immunogenicity, and combining multiple therapeutic modalities. Pairing optimized DNA-LNPs with ICI treatment offers the potential for widely applicable cancer immunotherapies.

## Methods

### Sex as a biological variable.

For the genetically engineered mouse model of HCC and for the nonhepatic tumor models, female mice were used. For the chemically-induced HCC model, male mice were used as it is known that male mice show higher rates of tumor formation after DEN treatment (68). For other models, female mice were chosen since they are less aggressive and can be housed together, reducing cost and variability while improving data consistency. Also, female mice have more stable body weights, which facilitates tracking tumor burden.

### LNP formulation and characterization.

Two noncoding plasmids without promoter sequences were used for LNP formulations: DNA (5.1 kb CpG-free plasmid from GenScript) and DNA2 (1.3 kb Nanoplasmid with 16 CpG from Aldevron) (sequence information is available in the appendix in the supplemental materials). Two ionizable lipids were used for LNP formulation: lipid 9 synthesized following the previous protocol (69) and branched CKK-E12.

LNPs were formulated following previously published protocols (21). In brief, lipids (in absolute ethanol) and DNA (in 50 mM citrate buffer, pH 3) solutions underwent microfluidic mixing using the NanoAssemblr Ignite microfluidic device (Cytiva) at a 3:1 volume ratio. The mass ratio of ionizable lipid to DNA was 10:1. For lipid 9 LNPs (LNP), a mixture of ionizable lipid, distearoylphosphatidylcholine, cholesterol, and C14-PEG 2000 lipids at a molar ratio of 50:10:38.5:1.5 was diluted in ethanol. For branched CKK-E12 LNP (LNP2), a mixture of ionizable lipid, 1,2-dioleoyl-sn-glycero-3-phosphoethanolamine, cholesterol, and C14-PEG 2000 lipids at a molar ratio of 35:16:46.5:2.5 was diluted in ethanol. Empty LNPs were formulated as described previously (70). Briefly, they were formulated using the identical composition and amount of lipids (18.2 μg total lipids per 1 μg payload equivalent) as their corresponding DNA-LNP, excluding the payload. For administration, both DNA-LNPs and empty LNPs were diluted the same way, ensuring equivalent lipid content and final volume.

LNPs were loaded into Slide-A-Lyzer Dialysis Cassettes, 20 kDa MWCO (Thermo Fisher Scientific) and dialyzed in PBS (pH 7.4, no calcium, no magnesium) for 2 hours at room temperature, followed by overnight dialysis at 4°C. After dialysis, LNPs were concentrated using Amicon ultra centrifugal filters, 50 kDa MWCO (Sigma-Aldrich), syringe-filtered, and stored in 20% glucose-PBS solutions at –80°C.

LNP particle size and polydispersity index (PDI) were measured using Zetasizer Nano ZS (Malvern Panalytical). DNA encapsulation efficiency was measured using the RiboGreen assay (Thermo Fisher Scientific) as previously described (21). The size of DNA-LNPs ranged from 73 to 97 nm and DNA2-LNPs ranged from 58 to 77 nm. The size of empty LNPs was 62 nm, comparable to that of LNPs containing payloads. The PDI was below 0.2 in all cases, and the encapsulation efficiency was above 80%.

### Plasmid constructs and DNA preparations for HCC induction.

Transposon constructs containing genes encoding human N90-β-catenin and human c-MET were obtained from Addgene (pT3-N90-β-catenin 31785 and pT3-EF1a-cMET 31784). Plasmid encoding HSB2 was a gift from Tushar Patel (Mayo Clinic, Jacksonville, Florida). The *Gaussia* luciferase sequence was synthesized by GenScript and cloned into a pT3 backbone to obtain pT3-GLuc plasmid. The pT3 empty vector control was generated by digesting pT3-N90-β-catenin with EcoRV and allowing recircularization of the backbone. DNA preparations were made by using endotoxin-free maxi or mega prep kits (Invitrogen), and the identity of each plasmid was confirmed by enzymatic digestion and Sanger sequencing.

### Animals.

For HCC models, 6-week-old female or 2-week-old male C57BL/6J (WT) mice were purchased from Charles River Laboratories and were maintained at the animal facility of the School of Medicine at the University of Barcelona (register B9900020) in a specific pathogen–free facility in accordance with an approved animal care and use protocol. For nonhepatic tumor models, 6- to 9-week-old female C57BL/6 mice were purchased from Shanghai Lingchang Biotechnology.

### HCC cancer models and treatments.

A genetically engineered mouse model of HCC was generated by delivering oncogene constructs to hepatocytes by using the hydrodynamic transfection technique. Briefly, pT3-EF1a–c-MET (20 μg), pT3-EF1a–ΔN90-β-catenin (20 μg), pT3-Gluc (4 μg), and HSB2 (4.4 μg) were diluted in saline solution (Braun) in a total volume of 1 mL per mouse. This volume was adjusted to 10% of each mouse’s body weight at the moment of administration (approximately 2 mL) and injected over 5–7 seconds into the lateral tail veins. For indicated experiments, control mice received pT3 empty vector (40 μg) instead of the plasmids encoding for the oncogenes. Three or 4 weeks after the hydrodynamic injection, as indicated, mice were treated with a single i.v. injection of either vehicle (PBS), DNA-LNPs, or empty LNPs at indicated doses (0.2, 1, or 10 μg) in a total volume of 100 μL. In some experiments, anti-mouse PD-L1 antibodies (InVivoMAb, BE0101 clone 10F.9G2, Bio X Cell) were administered via i.p. injection twice per week for a total of 2 weeks (4 doses) at a 200 μg/mouse dose in a total volume of 100 μL starting at day 1 after DNA-LNP dosing. In some experiments, anti-mouse CD8A antibodies (InVivoMAb, BE0061 clone 2.43, Bio X Cell) were i.p. administered on days –2, 1, 4, and 7 after DNA-LNP dosing at a dose of 200 μg/mouse in a total volume of 100 μL starting at day 1 after DNA-LNP dosing. At the endpoint, livers were harvested, and liver weight was measured in order to represent liver/body weight ratio as a measure of tumor burden. Survival curves were graphed considering the time point when the mice needed to be euthanized due to external signs of distress or tumor burden according to the ethical protocols. At indicated time points, mouse blood was collected through submandibular bleeds in serum separator tubes (BD Microtainer coagulation activator and SST gel, 365968, Becton Dickinson), and then serum was recovered by centrifuging tubes for 5 minutes at 10,000*g*. Serum luciferase levels were determined using the Pierce Gaussia Luciferase Glow assay kit (Thermo Fisher Scientific) according to the manufacturer’s indications. Briefly, 5 μL of serum was plated on a 96-well flat-bottom white polystyrene assay plate (Costar) and mixed with 50 μL of the substrate reagent. Luminescence measurements were acquired after 10 minutes using a Synergy HT plate reader (BioTek).

In the chemically induced model, HCC was induced by a single i.p. administration of 25 mg/kg of DEN to 2-week-old male C57BL/6J mice. Mice were treated with an i.v. injection of noncoding DNA-LNP 36 weeks after DEN administration, based on previous studies typically administering therapeutic interventions between 8 and 10 months after DEN induction (27, 71). No external clinical signs of diseases were shown during the 36 weeks of HCC induction. Six weeks after DNA-LNP treatment, mice were euthanized. At the endpoint, liver weight was measured, and tumor nodules were counted.

### Nonhepatic cancer models and treatments.

C1498 (American Type Culture Collection) and B16-F10 tumor cells (Shanghai Institutes for Biological Sciences) were maintained with DMEM supplemented with 10% FBS at 37°C and 5% CO_2_. The cells in the exponential growth phase were harvested for tumor inoculation.

For the C1498 AML model, mice were i.v. inoculated with 1 × 10^6^ cells in 0.1 mL of PBS. Three days after inoculation (day 0), mice were randomized based on body weight.

For the s.c. B16-F10 melanoma model, mice were s.c. inoculated at the right lower flank region with 2 × 10^5^ cells in 0.1 mL of PBS. For the B16-F10 melanoma model with tumors on both sides, the left lower flank region of the mice was also inoculated with 1 × 10^5^ cells in 0.1 mL of PBS. Next, 6–8 days after inoculation (day 0), mice were randomized based on tumor volume of the right flank. Tumor volume was measured every 3 days using a caliper: volume = (length × width^2^) / 2. Individual mice were euthanized when the total tumor volume exceeded 3,000mm^3^.

For the B16-F10 lung metastasis model, mice were i.v. inoculated with 1 × 10^5^ cells in 0.1 mL of PBS. Mice were randomized based on body weight 5 days after inoculation (day 0). Lung tissues were collected on day 13, and the tumor nodules on the tissue surface were counted in a blinded fashion.

Mice received various treatments including vehicle (20% glucose in PBS), DNA-LNPs, ADU-S100, and antibodies. The following dosing materials were purchased for the study: ADU-S100 from MedChemExpress and InVivoMAb rat IgG2a isotype control (BE0089, clone 2A3), anti-mouse CD8α (BE0061, clone 2.43), anti-mouse NK1.1 (BE0036, clone PK136), and anti-mouse PD-L1 (BE0101, clone 10F.9G2) from BioXCell. Mice received 100 μL volume for i.v. and i.p. dosing and 50 μL for intratumoral dosing. Mice in the bilateral tumor experiment received 100 μL intratumorally.

Animals were checked daily for morbidity and mortality. Body weight loss was measured at 1 day after dosing in indicated studies and twice per week thereafter. A supplemental diet gel was supplied to all mice in the same group if more than 15% of body weight loss was observed. Animals were euthanized when the humane endpoints were met or when more than 20% of body weight loss was observed.

### Cytokine analysis.

Serum cytokine levels in the HCC models were analyzed at indicated time points after treatment by using a multiplex assay (LegendPlex mouse inflammation panel (13-flex), BioLegend) according to the manufacturer’s indications. Data were acquired using a BD LSR Fortessa 5L and analyzed with Qognit software (BioLegend). Serum cytokine levels in the nonhepatic tumor models were analyzed by Luminex according to the manufacturer’s instructions (ProcartaPlex Mouse and Rat Mix & Match, MAN0025393, Thermo Fisher Scientific).

### RNA extraction and gene expression analysis.

Livers were harvested, snap-frozen on dry ice, and kept at –80°C for later manipulations. Frozen livers were disrupted and homogenized using TissueLyser LT technology (QIAGEN). Briefly, 2 mL microcentrifuge tubes containing 1 stainless steel bead of 5 mm mean of diameter (QIAGEN) were placed on dry ice for at least 15 minutes. Approximately 30 mg of liver tissue was transferred to the precooled tubes and incubated for another 15 minutes on dry ice. The tubes were then incubated at room temperature for 2 minutes, and 300 μL of RLT lysis buffer was added. The TissueLyser LT was then operated for 5 minutes at 50 Hz, and RNA was isolated by using the RNeasy mini kit (QIAGEN). cDNA was prepared from 1 μg of RNA by using the high-capacity RNA-to-cDNA kit (Applied Biosystems), and gene expression levels were determined using qPCR with TaqMan probes *MET* (Hs01565584_m1), *CTNNB1* (bCAT, Hs00355045_m1), and *Cd8a* (Mm01182107_g1) according to the manufacturer’s (Applied Biosystems) indications. *Actb* (Mm02619580_g1) was used as a housekeeping control. A 7900HT Fast Real-Time PCR System was used (Applied Biosystems). Gene expression analysis was performed using the PanCancer_Mouse_IO_360_Panel from NanoString Technologies. Liver RNA was extracted as described above. Samples were prepared according to the manufacturer’s protocols. Cartridges were run on the nCounter SPRINT Profiler. Gene expression levels were normalized against the housekeeping genes, and data analysis was conducted using the nSolver 4.0 software (NanoString Technologies). Immune cell populations were defined by the following signatures: T cells (*Cd3d*, *Cd3e*, *Cd3g*, *Cd6*, *Sh2d1a*, and *Trat1*); cytotoxic cells (*Prf1*, *Klrb1*, *Klrd1*, *Klrk1*, *Nkg7*, *Gzma*, *Gzmb*, and *Ctsw*); exhausted Cd8 (*Cd244*, *Eomes*, *Lag3*, and *Ptger4*); B cells (*Blk*, *Cd19*, *Fcrlb*, *Ms4a1*, *Pnoc*, *Spib*, *Tcl1*, and *Tnfrsf17*); NK cells (*Ncr1* and *Xcl1*); DCs (*Ccl2*, *Cd209e*, and *Hsd11b1*); mast cells (*Cpa3*, *Hdc*, and *Ms4a2*); neutrophils (*Ceacam3*, *Csf3r*, *Fcgr4*, and *Fpr1*); macrophages (*Cd163*, *Cd68*, *Cd84*, and *Ms4a4a*); and CD45 (*Ptprc*). Volcano plots were done in R version 4.3.2. GSEA was performed using the fgsea package (v.1.28.0) in R.

### IHC.

Liver tissues were fixed with 10% normal buffered formalin overnight and then transferred to 70% ethanol. Paraffin blocks were made from the fixed tissues, and slides cut from the blocks were stained for catenin β-1 (dil 1:75, 8480, clone D10A8), MET (dil 1:100, 8198, clone D1C2), mouse CD8A (dil 1:500, 98941, clone D4W2Z), and mouse MKI67 (dil 1:800, 12202, clone D3B5). All antibodies were purchased from Cell Signaling Technology. Paraffin blocks, slides, and IHC staining were performed by the BioBank at IDIBAPS/Hospital Clínic. Digital scan and image analysis of IHC was performed at the Histopathology Facility at the Institute for Research in Biomedicine. For the B16-F10 lung metastasis model, lung tissues were collected on day 13, formalin-fixed and paraffin-embedded, and sectioned to 4 μm thickness. Tissue sections were treated with BOND epitope retrieval solution 2 (Leica Biosystems) and stained with CD8 antibody from eBioscience (14-0195-82, clone 4SM16) and BOND polymer refine red detection kit (Leica Biosystems). Slides were imaged using Zeiss Axio Scan Z1. CD8-expressing cells were quantified using HALO v3.5 (Indica Labs). Lobes of the lung were marked as a tissue area, and the tumor area with black pigments was annotated by hand in a blinded fashion and quantified using Halo v3.5.

### Flow cytometry.

Dissected B16-F10 melanoma tissue was homogenized using a mouse tumor dissociation kit and gentleMACS Octo dissociator with heaters (Miltenyi Biotec) following the manufacturer’s instructions. Cells were stained with ViaStain AOPI staining solution and counted using Cellaca MX high-throughput cell counter (Revvity). The cell suspension was stained with Zombie Aqua fixable viability kit (BioLegend) for 10 minutes at room temperature and then washed twice with PBS. Cells were then preincubated with anti-mouse CD16/32 for 5 minutes on ice and labeled with fluorophore-conjugated antibodies in cell staining buffer (BioLegend) for 15 minutes on ice. Cells were then fixed and permeabilized using Cyto-Fast Fix/Perm buffer set, intracellularly stained with fluorophore-conjugated antibodies for 15 minutes at room temperature, and washed 3 times. The following antibodies from BioLegend were used: CD16/32 for blocking (101301, clone 93), CD45.2 (103127, clone 30-F11), CD3 (100217, clone 17A2), CD4 (100527, clone RM4-5), CD8 (100707, clone 53-6.7), NK1.1 (108709, clone PK136), CD69 (104527, clone H1.2F3), and Granzyme B (515403, clone GB11). Stained cells were analyzed with BD LSR Fortessa and FlowJo software.

### Statistics.

Statistical significance analyses were performed using GraphPad Prism. Survival significance was assessed using Kaplan-Meier/Mantel-Cox log-rank tests. Statistical differences between experimental groups were determined by 1-way ANOVA with Tukey’s multiple-comparison test or 2-tailed Student’s *t* test as indicated in the figure legends. A *P* value less than 0.05 was considered significant.

### Study approval.

All animal procedures were approved by the Ethics Committee for Animal Experimentation (CEEA) of the University of Barcelona and Generalitat de Catalunya or the IACUC of Crown Bioscience. The care and use of animals were conducted in accordance with the regulations of the Association for Assessment and Accreditation of Laboratory Animal Care.

### Data availability.

NanoString transcriptomics data have been deposited in the Zenodo database (https://doi.org/10.5281/zenodo.17378468). Values for all data in graphs are reported in the Supporting Data Values file. Additional data will be made available upon request.

## Author contributions

SY and ARG designed and performed experiments, analyzed and interpreted the data, and wrote the manuscript. SY is listed first based on the extent of her contributions to the final figures. JC and GC performed in vivo experiments. MGA, SC, MAL, and MAM performed experiments. TLJ assisted with gene expression data analysis. MNY designed noncoding plasmid constructs. RZ, XMA, AN, MCW, SMA, SG, and PJC supervised the project, including design of experiments, data analysis and interpretation, and manuscript writing.

## Funding support

- Spark Therapeutics Inc.
- Non-clinical Research Operations, Histology, and Medical Communications teams at Spark Therapeutics.
- Spanish Ministry of Science and Innovation under a Ramon y Cajal grant (RYC2018-024442-I) to SG.
- Spanish Association Against Cancer (INVES222988RODR) to ARG.

## Supplementary Material

Supplemental data

Supporting data values

## Version 1 03/23/2026

Electronic publication
