# Supplementary material for: DNA delivered by lipid nanoparticles induces CD8+ T cell–dependent antitumor responses and enhances anti–PD-L1 therapy
Source: JCI Insight. 2026 Mar 23;11(6):e197404. doi: 10.1172/jci.insight.197404 (PMC13043106; doi:10.1172/jci.insight.197404)
Supplement: Supplemental data [file jciinsight-11-197404-s080.pdf]

Supplemental Material

DNA delivered by lipid nanoparticles induces $CD8^+$ T cell-dependent antitumor responses and enhances anti-PD-L1 therapy.

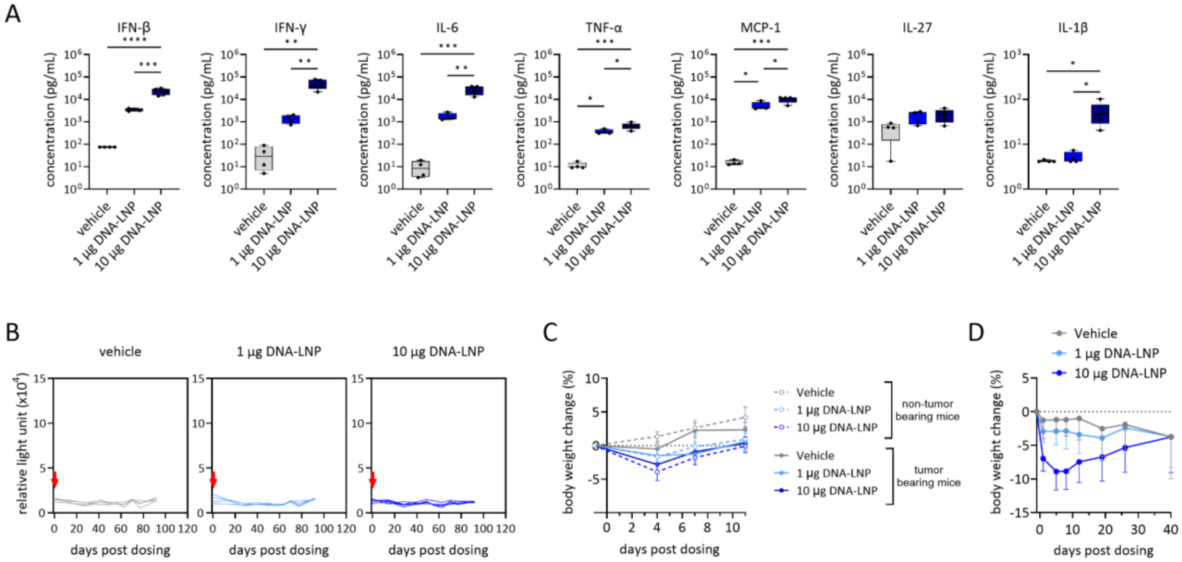

Supplemental Figure 1. Responses to DNA-LNP in mice with or without HCC. (A-C) As a control of the genetically-induced HCC model, mice without HCC induction was developed by HDTVI of plasmids encoding GLuc, HSB2, and control plasmids without oncogenes. 3 weeks after HDTVI, mice received a single dose of DNA-LNP intravenously. (A) Serum cytokine levels 4 hours after dosing. (B) Absence of tumor growth indicated by serum GLuc activity. (C) Body weight change overtime in the non-tumor bearing mice or HDTVI-induced HCC model that received a single dose of DNA-LNP intravenously 3 weeks after HDTVI of plasmids. (D) Body weight change overtime in the chemically-induced HCC model. Min-to-Max whiskers are shown in the Box and whiskers plot. Mean with SD is shown in dot or line graphs. Data were analyzed by one-way ANOVA with Tukey's multiple comparisons test. * p<0.05, ** p<0.01, *** p<0.001, and **** p<0.0001.

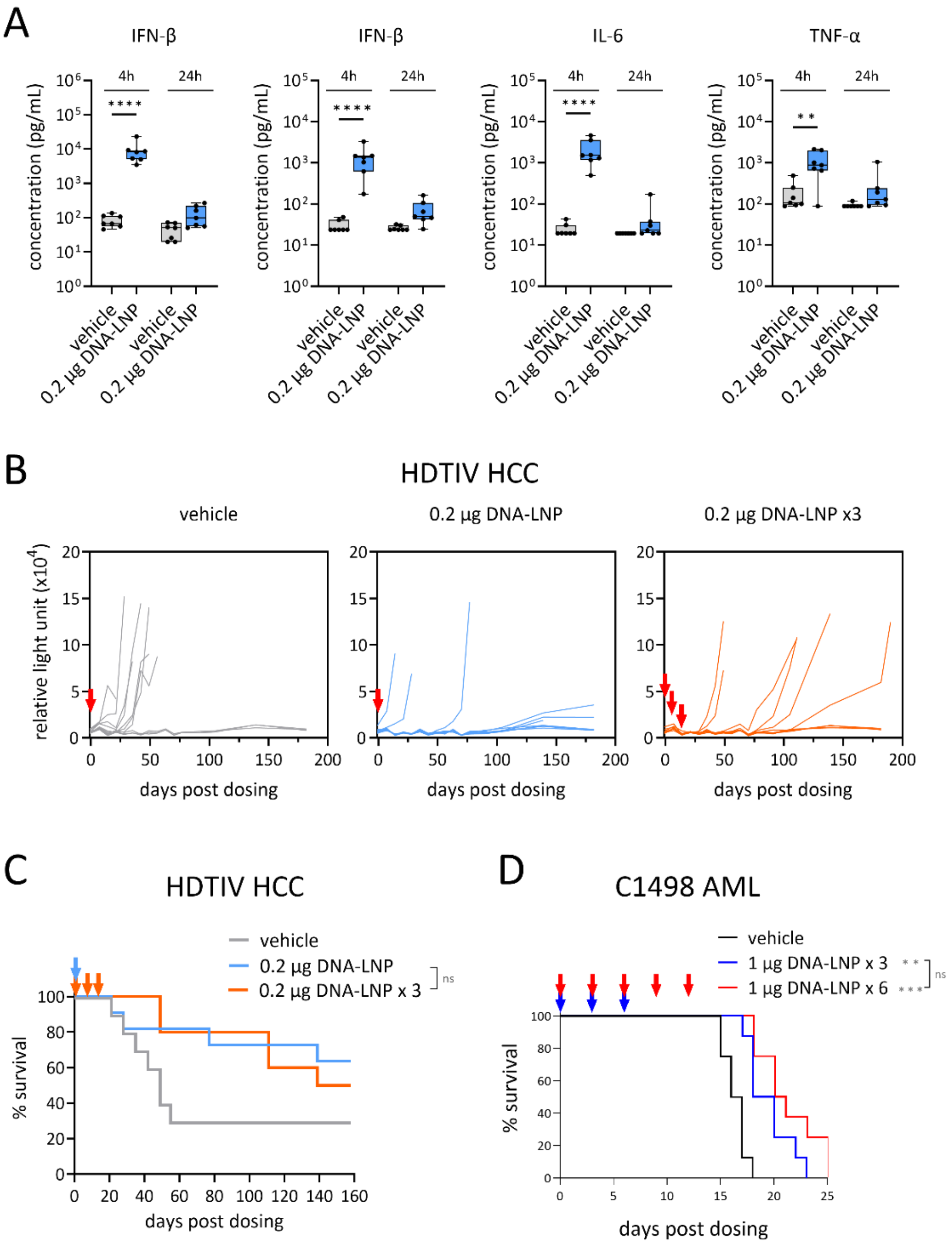

Supplemental Figure 2. DNA-LNP induces transient cytokine elevation, and extending the repeated dosing showed no additional antitumor effect. (A-C) HCC was induced by HDTVI of plasmids encoding GLuc, c-Met, β-Catenin, and HSB2. 3 weeks after HCC induction, mice received a single dose or three weekly doses of DNA-LNP. (A) Serum cytokine levels 4 and 24 hours after the first dosing. (B) Tumor growth indicated by serum GLuc activity. (C) Survival of HCC-bearing mice. (D) Survival curves of C1498 AML tumor-bearing mice (n=8). Mice were intravenously dosed with 1 µg of DNA-LNP at indicated time. Vehicles were dosed 3 times, 3 days apart. Survival data were analyzed by log rank (Mantel-Cox) tests, and other data were analyzed by one-way ANOVA with Tukey's multiple comparisons test. P-value indicates comparison to the vehicle-treated group unless otherwise indicated. * $p<0.05$, ** $p<0.01$, *** $p<0.001$, and **** $p<0.0001$.

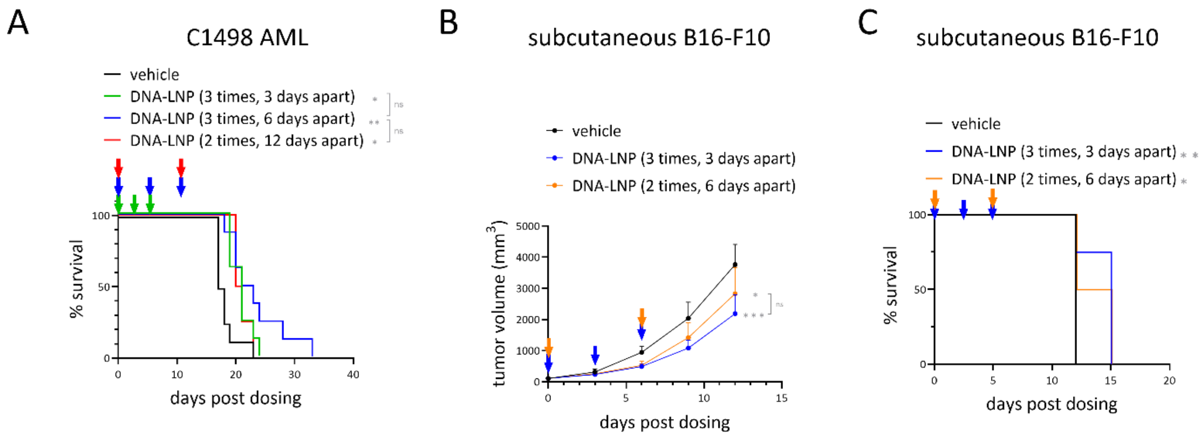

Supplemental Figure 3. Antitumor effect is maintained with a reduced frequency of DNA-LNP administration. (A) Survival curves of C1498 AML tumor-bearing mice (n=8). Mice were intravenously dosed with 1 µg of DNA-LNP at indicated time. Vehicles were dosed 3 times, 3 days apart. (B-C) Mice bearing B16-F10 tumors (n=8) at one side received treatments intratumorally at indicated timepoints. Vehicles were dosed 3 times, 3 days apart. (B) Tumor growth curve. Data were analyzed at day 12 post-dosing using one-way ANOVA with Tukey's multiple comparisons test. (C) Survival curve. Survival data were analyzed by log rank (Mantel-Cox) tests. P-value indicates comparison to the vehicle-treated group unless otherwise indicated. * $p<0.05$, ** $p<0.01$.

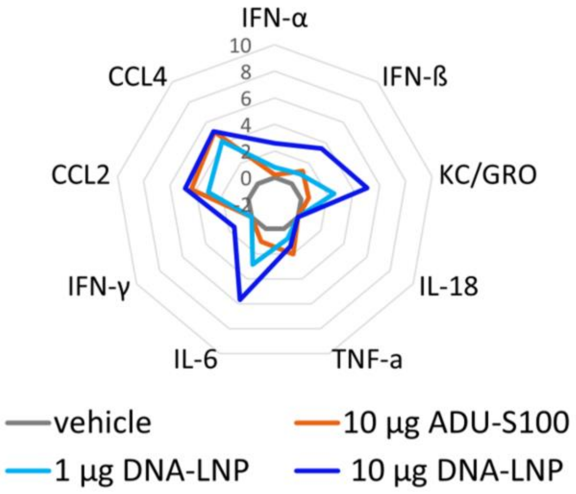

Supplemental Figure 4. DNA-LNP induces cytokines in the subcutaneous B16-F10 melanoma model. Serum cytokine levels 4h after the first dosing. Average values (n=8) are represented as log2 fold-increase over vehicle. B16-F10 tumors were inoculated to both sides of mice (n=8) and intratumorally dosed with treatments at one tumor site (local) at day 0, 3, and 6.

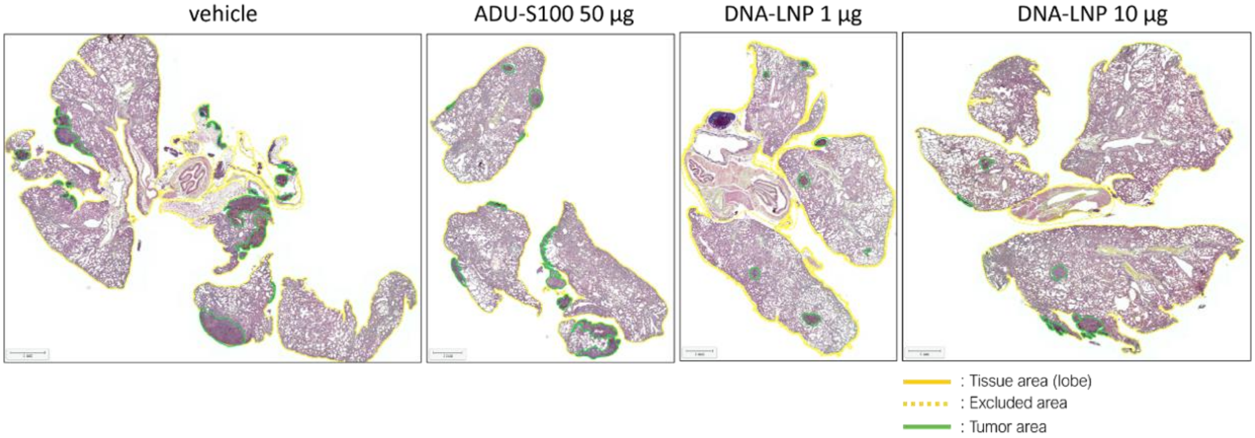

Supplemental Figure 5. Representative images of IHC lung sections from the B16-F10 lung metastasis model. Mice with B16-F10 lung metastasis were dosed intravenously at day 0, 3, and 6, and liver sections were analyzed at day 13.

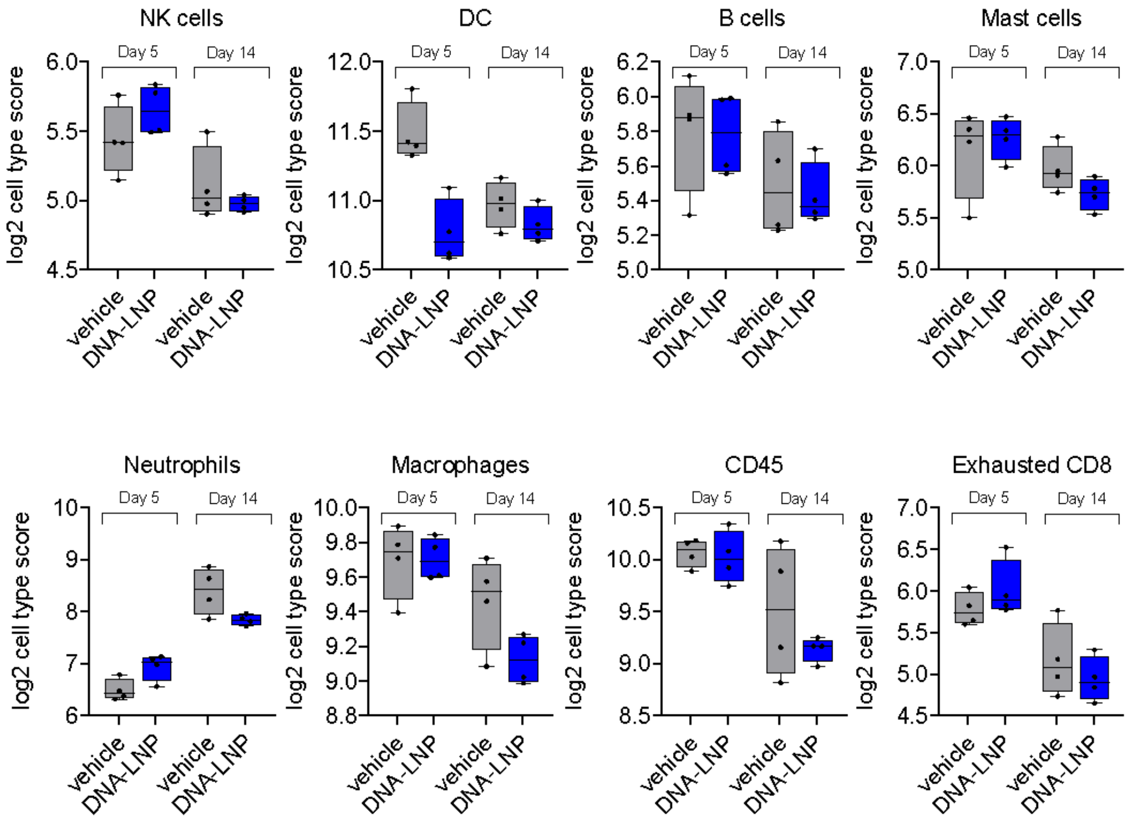

Supplemental Figure 6. Nanostring analysis of immune cell infiltration to the liver in the HDTVI-HCC model. HCC was induced by HDTVI of plasmids encoding GLuc, c-Met, β-Catenin, and HSB2. 3 weeks after HCC induction, mice were treated with 1 µg of DNA-LNP intravenously. Livers were harvested and analyzed at days 5 and 14 post-treatment, and cell type profiling was done by Nanostring. Min-to-Max whiskers are shown in the Box and whiskers plot. Differences were not significant when analyzed by one-way ANOVA with Tukey's multiple comparisons test.

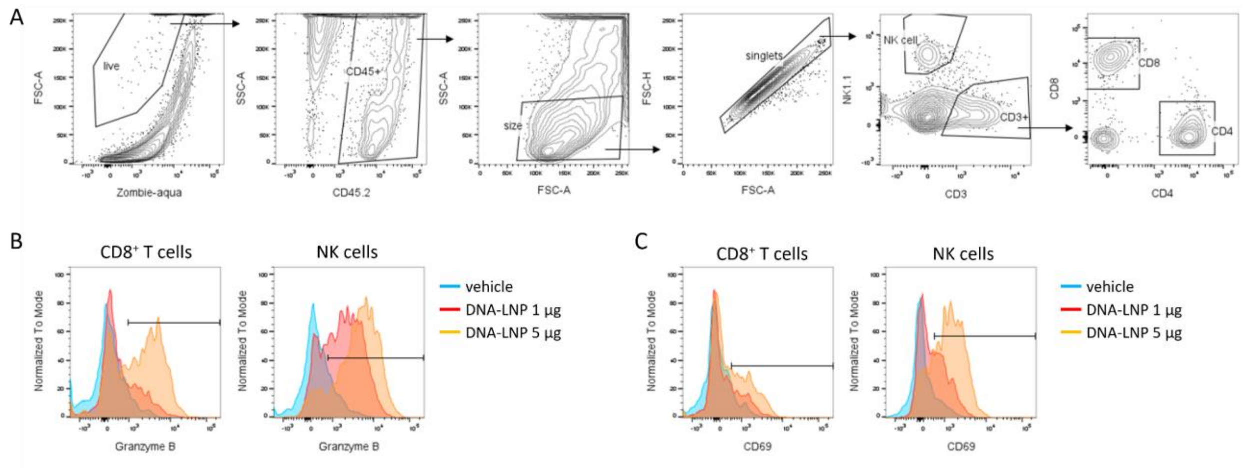

Supplemental Figure 7. Flow cytometry analysis of immune cells from subcutaneous B16-F10 melanoma model. Subcutaneous B16-F10 tumor-bearing mice were intratumorally dosed with DNA-LNP at day 0 and 3. Tumors were collected at day 7 and analyzed using flow cytometry. (A) Gating scheme. (B) Representative plots of Granzyme B^{+} CD8^{+} T cells or NK cells. (G) Representative plots of CD69^{+} CD8^{+} T cells or NK cells.

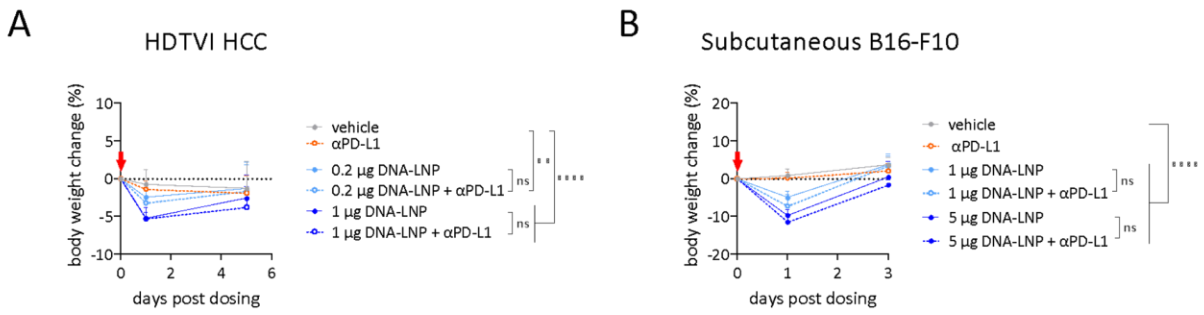

Supplemental Figure 8. Anti-PD-L1 combination did not exacerbate body weight loss induced by DNA-LNP. (A) Body weight loss in HCC-bearing mice after DNA-LNP and anti-PD-L1 treatment. Genetically-induced HCC models were intravenously dosed with the indicated doses of DNA-LNP 4 weeks after HDTVI. 200 µg of anti-PD-L1 was dosed intraperitoneally at day 1, 5, 8, and 11. (B) Body weight loss in melanoma-bearing mice after DNA-LNP and anti-PD-L1 treatment. B16-F10 tumor-bearing mice were intraperitoneally dosed with 250 µg of anti-PD-L1 and intratumorally dosed with the indicated doses of DNA-LNP at day 0, 3, and 6. Mean with SD is shown. P-values were analyzed by one-way ANOVA with Tukey's multiple comparisons test. ** p<0.01, **** p<0.0001, and ns (non-significant).

Appendix

Full sequence of DNA
>TTAATTAAAATTATCTCTAAGGCATGTGAACTGGCTGTCTTGGTTTTCATCTGTACTTCATCTGCTACCTCTGTGACCTGAAACA
TATTTATAATTCCATTAAGCTGTGCATATGATAGATTTATCATATGTATTTTCCTTAAAGGATTTTTGTAAGAACTAATTGAATTG
ATACCTGTAAAGTCTTTATCACACTACCCAATAAATAATAAATCTCTTTGTTCAGCTCTCTGTTTCTATAAATATGTACCAGTTTTA
TTGTTTTTAGTGGTAGTGATTTTATTCTCTTTCTATATATATACACACACATGTGTGCATTCATAAATATATACAATTTTTATGAAT
AAAAAATTATTAGCAATCAATATTGAAAACCACTGATTTTTGTTTATGTGAGCAAACAGCAGATTAAAAGGAATTCCTGCAGGG
CCCACCTAGGATGCATAGTACTAGGATCCAACATGTAACTAGTAGCATGCAAAGCTTAGAATTGTACTAACCTTCTTCTCTTTCC
TCTCCTGACAGGTTGGTGTACAGTAGCTTCCACCATGCAGAGGGTGAACATGATCATGGCTGAGAGCCCTGGCCTGATCACCA
TCTGCCTGCTGGGCTACCTGCTGTCTGCTGAATGTACAGGTTTGTTTCCTTTTTTATAATACATTGAGTATGCTTGCCTTTTAGAT
ATAGAAATATCTGATTCTGTCTTCTTCACTAAATTTTGATTACATGATTTGACAGCAATATTGAAGAGTCTAACAGCCAGCACCC
AGGTTGGTAAGTACTGGTTCTTTGTTAGCTAGGTTTTCTTCTTCTTCACTTTTAAAACTAAATAGATGGACAATGCTTATGATGC
AATAAGGTTTAATAAACACTGTTCAGTTCAGTATTTGGTCATGTAATTCCTGTTAAAAAACAGTCATCTCCTTGGTTTAAAAAAA
TTAAAAGTGGGAAAACAAAGAAATAGCAGAATATAGTGAAAAAAAATAACCACAGTATTTTTGTTTGGACTTACCACTTTGAA
ATCAAATTGGGAAACAAAAGCACAAACAGTGGCCTTATTTACACAAAAAGTCTGATTTTAAGATATGTGACAATTCAAGGTTTC
AGAAGTATGTAAGGAGGTGTGTCTCTAATTTTTTAAATTATATATCTTCAATTTAAAGTTTTAGTTAAAACATAAAGATTAACCT
TTCATTAGCAAGCTGTTAGTTATCACCAAAGCTTTTCATGGATTAGGAAAAAATCATTTTGTCTCTATCTCAAACATCTTGGAGT
TGATATTTGGGGAAACACAATACTCAGTTGAGTTCCCTAGGGGAGAAAAGCAAGCTTAAGAATTGACACAAAGAGTAGGAAG
TTAGCTATTGCAACATATATCACTTTGTTTTTTCACAACTACAGTGACTTTATTTATTTCCCAGAGGAAGGCATACAGGGAAGAA
ATTATCCCATTTGGACAAACAGCATGTTCTCACAGTAAGCACTTATCACACTTACTTGTCAACTTTCTAGAATCAAATCTAGTAG
CTGACAGTACCAGGATCAGGGGTGCCAACCCTAAGCACCCCCAGAAAGCTGACTGGCCCTGTGGTTCCCACTCCAGACATGAT
GTCAGCTGTGAAATCCACCTCCCTGGACCATAATTAGGCTTCTGTTCTTCAGGAGACATTTGTTCAAAGTCATTTGGGCAACCAT
ATTCTGAAAACAGCCCAGCCAGGGTGATGGATCACTTTGCAAAGATCCTCAATGAGCTATTTTCAAGTGATGACAAAGTGTGA
AGTTAAGGGCTCATTTGAGAACTTTCTTTTTCATCCAAAGTAAATTCAAATATGATTAGAAATCTGACCTTTTATTACTGGAATTC
TCTTGACTAAAAGTAAAATTGAATTTTAATTCCTAAATCTCCATGTGTATACAGTACTGTGGGAACATCACAGATTTTGGCTCCA
TGCCCTAAAGAGAAATTGGCTTTCAGATTATTTGGATTAAAAACAAAGACTTTCTTAAGAGATGTAAAATTTTCATGATGTTTTC
TTTTTTGCTAAAACTAAAGAATTATTCTTTTACATTTCAGTTTTTCTTGATCATGAAAATGCCAACAAAATTCTGAATAGACCAAA
GAGGTATAACTCTGGCAAGCTTGAAGAGTTTGTACAGGGGAATCTGGAGAGAGAGTGTATGGAAGAGAAGTGCAGCTTTGA
GGAAGCCAGAGAAGTGTTTGAAAATACAGAGAGAACAACTGAATTTTGGAAGCAGTATGTGGATGGTGATCAATGTGAGAGC
AATCCCTGCTTGAATGGGGGGAGCTGTAAAGATGATATCAACAGCTATGAATGTTGGTGTCCCTTTGGATTTGAGGGGAAAAA
CTGTGAGCTTGATGTGACCTGTAATATCAAGAATGGCAGGTGTGAGCAATTTTGCAAGAATTCTGCTGATAACAAAGTGGTCT
GTAGCTGCACTGAGGGATATAGGCTGGCTGAAAACCAGAAGAGCTGTGAACCTGCAGTGCCTTTTCCCTGTGGGAGAGTGTC
TGTGAGCCAAACCAGCAAGCTGACTAGGGCTGAAGCAGTCTTTCCTGATGTAGATTATGTGAATAGCACTGAGGCTGAGACAA
TCCTTGACAATATCACTCAGAGCACACAGAGCTTCAATGACTTCACCAGGGTGGTAGGAGGGGAGGATGCCAAGCCTGGGCA
GTTCCCCTGGCAGGTAGTGCTCAATGGAAAAGTGGATGCCTTTTGTGGAGGTTCAATTGTAAATGAGAAGTGGATTGTGACTG
CAGCCCACTGTGTGGAAACTGGAGTCAAGATTACTGTGGTGGCTGGAGAGCACAATATTGAGGAAACTGAGCACACTGAGCA
GAAGAGGAATGTGATCAGGATTATCCCCCACCACAACTACAATGCTGCTATCAACAAGTACAACCATGACATTGCCCTCCTGGA
ACTGGATGAACCCCTGGTCTTGAACAGCTATGTGACACCCATCTGTATTGCTGATAAAGAGTACACCAACATCTTCTTGAAATTT
GGGTCTGGATATGTGTCTGGCTGGGGCAGGGTGTTCCATAAAGGCAGGTCTGCCCTGGTATTGCAGTATTTGAGGGTGCCTCT
GGTGGATAGAGCAACCTGCTTGAGAAGCACCAAGTTTACAATCTACAACAATATGTTCTGTGCAGGGTTCCATGAAGGTGGTA
GAGACAGCTGCCAGGGAGATTCTGGGGGTCCCCATGTGACTGAGGTGGAGGGAACCAGCTTCCTGACTGGGATTATCAGCTG
GGGTGAGGAGTGTGCTATGAAGGGAAAGTATGGGATCTACACAAAAGTATCCAGATATGTGAACTGGATTAAGGAGAAAAC
CAAGCTGACTTGATAGCTAGCTGGCCAGACATGATAAGATACATTGATGAGTTTGGACAAACCACAACTAGAATGCAGTGAAA
AAAATGCTTTATTTGTGAAATTTGTGATGCTATTGCTTTATTTGTAACCATTATAAGCTGCAATAAACAAGTTAACAACAACAAT
TGCATTCATTTTATGTTTCAGGTTCAGGGGGAGGTGTGGGAGGTTTTTTAAAGCAAGTAAAACCTCTACAAATGTGGTATGGA
ATTCAGTCAATATGTTCACCCCAAAAAAGCTGTTTGTTAACTTGCCAACCTCATTCTAAAATGTATATAGAAGCCCAAAAGACAA
TAACAAAAATATTCTTGTAGAACAAAATGGGAAAGAATGTTCCACTAAATATCAAGATTTAGAGCAAAGCATGAGATGTGTGG
GGATAGACAGTGAGGCTGATAAAATAGAGTAGAGCTCAGAAACAGACCCATTGATATATGTAAGTGACCTATGAAAAAAATA
TGGCATTTTACAATGGGAAAATGATGGTCTTTTTCTTTTTTAGAAAAACAGGGAAATATATTTATATGTAAAAAATAAAAGGGA
ACCCATATGTCATACCATACACACAAAAAAATTCCAGTGAATTATAAGTCTAAATGGAGAAGGCAAAACTTTAAATCTTTTAGA
AAATAATATAGAAGCATGCCATCAAGACTTCAGTGTAGAGAAAAATTTCTTATGACTCAAAGTCCTAACCACAAAGAAAAGATT
GTTAATTAGATTGCATGAATATTAAGACTTATTTTTAAAATTAAAAAACCATTAAGAAAAGTCAGGCCATAGAATGACAGAAAA

TATTTGCAACACCCCAGTAAAGAGAATTGTAATATGCAGATTATAAAAAGAAGTCTTACAAATCAGTAAAAAATAAAACTAGA
CAAAAATTTGAACAGATGAAAGAGAAACTCTAAATAATCATTACACATGAGAAACTCAATCTCAGAAATCAGAGAACTATCATT
GCATATACACTAAATTAGAGAAATATTAAAAGGCTAAGTAACATCTGTGGCTTAATTAAAATCAGCAGTTCAACCTGTTGATAG
TATGTACTAAGCTCTCATGTTTAATGTACTAAGCTCTCATGTTTAATGAACTAAACCCTCATGGCTAATGTACTAAGCTCTCATG
GCTAATGTACTAAGCTCTCATGTTTCATGTACTAAGCTCTCATGTTTGAACAATAAAATTAATATAAATCAGCAACTTAAATAGC
CTCTAAGGTTTTAAGTTTTATAAGAAAAAAAAGAATATATAAGGCTTTTAAAGGTTTTAAGGTTTCCTAGCTTTAGTCCTGTTCC
TCAGCTACAAAATGGACACAATTTCCAGCAGGGTCTCTGAGGGCAAATTCCCTTCCCCAAGGTTGTTCACCAATTTCTGTCATG
GCTGGGCCAGAGGCATCCCTGAAATTTGTGCTGACTACTTCTGACCATTCTGCATAAAGCTCATCTAGGCCTCTGACCCAGACC
CAAGCAAGGGTGTTGTCAGGGACAACTTGGTCCTGAACTGCTGAGATGAAGAGGGTGACATCATCTCTGACAACACCAGCAA
AATCATCTTCAACAAAGTCTCTGGAGAATCCTAATCTGTCAGTCCAGAACTCTACAGCCCCTGCAACATCCCTTGCTGTGAGGA
CTGGGACTGCAGAAGTGAGTTTGGCCATGATGGCCCTCCTATAGTGAGTTGTATTATACTATGCAGATATACTATGCCAATGTT
TAATTGTCAACTACCTGTT

Partial sequence of DNA2

The following sequence, which does not include promoter, was inserted into Aldevron’s R6K/RNA-OUT/CpG-free BGHpA nanoplasmid backbone. The final product resulted in 1.3 kb plasmid with 16 CpG sequences.

>GCCTCACCGTTGACCCGCTTTCTGTCGCTGAACCTGCTGCTGCTGGGTGAGTCGATTATCCTGGGGAGTGGAGAAGCTAACTT
TGGCCGACTTCACTGTACAACCGCAGTAATACGGAATATAAATGACCAAGTTCTCTTCGTTGACAAAAGACAGCCTGTGTTCGA
GGATATGACTGATATTGATCAAAGTGCCAGTGAACCCCAGACCAGACTGATAATATACATGTACAAAGACAGTGAAGTAAGA
GGACTGGCTGTGACCCTCTCTGTGAAGGATAGTAAAATGTCTACCCTCTCCTGTAAGAACAAGATCATTTCCTTTGAGGAAATG
GATCCACCTGAAAATATTGATGATATACAAAGTGATCTCATATTCTTTCAGAAACGTGTTCCAGGACACAACAAGATGGAGTTT
GAATCTTCACTGTATGAAGGACACTTTCTTGCTTGCCAAAAGGAAGATGATGCTTTCAAACTCATTCTGAAAAAAAAGGATGAA
AATGGGGATAAATCTGTAATGTTCACTCTCACTAACTTACATCAAAGTTAG
